# Joint Fairness Model with Applications to Risk Predictions for Under-represented Populations

**Published:** 2021-05-10

**Authors:** Hyungrok Do, Shinjini Nandi, Preston Putzel, Padhraic Smyth, Judy Zhong

**Affiliations:** 1 Department of Population Health, New York University Grossman School of Medicine, New York, NY 10016, USA; 2 Department of Mathematical Sciences, Montana State University, Bozeman, MT 59717, USA; 3 Department of Computer Science, University of California, Irvine, CA 92697, USA

**Keywords:** algorithmic fairness, algorithmic bias, joint estimation, under-represented population

## Abstract

Under-representation of certain populations, based on gender, race/ethnicity, and age, in data collection for predictive modeling may yield less-accurate predictions for the under-represented groups. Recently, this issue of fairness in predictions has attracted significant attention, as data-driven models are increasingly utilized to perform crucial decision-making tasks. Methods to achieve fairness in the machine learning literature typically build a single prediction model subject to some fairness criteria in a manner that encourages fair prediction performances for all groups. These approaches have two major limitations: i) fairness is often achieved by compromising accuracy for some groups; ii) the underlying relationship between dependent and independent variables may not be the same across groups. We propose a Joint Fairness Model (JFM) approach for binary outcomes that estimates group-specific classifiers using a joint modeling objective function that incorporates fairness criteria for prediction. We introduce an Accelerated Smoothing Proximal Gradient Algorithm to solve the convex objective function, and demonstrate the properties of the proposed JFM estimates. Next, we presented the key asymptotic properties for the JFM parameter estimates. We examined the efficacy of the JFM approach in achieving prediction performances and parities, in comparison with the Single Fairness Model, group-separate model, and group-ignorant model through extensive simulations. Finally, we demonstrated the utility of the JFM method in the motivating example to obtain fair risk predictions for under-represented older patients diagnosed with coronavirus disease 2019 (COVID-19).

## 1 Introduction

### 1.1 Applied Context

The issue of making fair predictions has attracted significant attention recently in machine learning as a critical issue in the application of data-driven models. Though machine learning models are increasingly utilized to perform crucial decision-making tasks, recent evidence reveals that many carefully designed algorithms learn biases from the underlying data and exploit these inequalities when making predictions. For example, large systematic biases in prediction performance have been detected for machine learning models in areas such as recidivism prediction relative to race [Angwin et al., 2016], ranking of job candidates relative to gender [Lahoti et al., 2018] and face recognition relative to both race and gender [Ryu et al., 2018, Buolamwini and Gebru, 2018]. There is an emerging recognition that such biases are also likely to be a significant issue in data-derived predictive models in healthcare [Char et al., 2018]. Data obtained through clinical trials are often biased and not representative of racial/ethnic minority groups and/or people over 75 with multiple chronic conditions [Gianfrancesco et al., 2018], a phenomenon which has appeared in studies of cancer incidence and mortality [Murthy et al., 2004], cardiovascular diseases [Sardar et al., 2014] and diabetes [Chow et al., 2012], etc. Biased representation of different populations in biomedical studies limits the benefits that can be potentially achieved for these communities.

One motivating example is to predict mortality for patients infected with coronavirus disease 2019 (COVID-19). As of January 23 2021, COVID-19 has infected more than 96 million people globally, accounting for more than 2 million known deaths. Older patients are particularly vulnerable to severe outcomes and death due to COVID-19. The Centers for Disease Control and Prevention (CDC) reported that the fatality rate was 18.8% for patients older than 80 years whereas the overall fatality rate is estimated at up to 5% for all patients [Kompaniyets et al., 2021]. This difference in survival highlights an urgent need for risk stratification of older patients with COVID-19 based on routine clinical assessments. However, most COVID-19 studies have not been stratified by age groups [Tehrani et al., 2021]. Thus, when a risk prediction equation generated from the general population was applied to older patients with COVID-19, the model predicted high-risk scores overall due to their older age, higher prevalence of comorbidities and more laboratory abnormalities. This resulted in insufficient and unfair risk stratification for these patients as not all older patients are at the same risk of death from COVID-19 [Tehrani et al., 2021].

### 1.2 Existing Approaches

Methods to address fairness in the machine learning literature typically begin with a formal probabilistic definition of fairness. In the context of risk prediction, predictive fairness at the group level means that a risk prediction model has performance characteristics (based on accuracy, ranking, calibration) that are relatively independent of group memberships. For example, if the false positive rate for a classification model is defined as *P*(*ŷ* = 1|*y* = 0), where *ŷ* is the model’s prediction, then enforcing equality with respect to a particular binary group indicator variable *G* can be stated as requiring the two predictive distributions *P*(*ŷ* = 1|*G* = 1, *y* = 0) and *P*(*ŷ* = 1|*G* = 0, *y* = 0) to be as close as possible. Other definitions include demographic parity [Calders et al., 2009], equalized odds or equal opportunities [Hardt et al., 2016], disparate treatment, impact and mistreatment [Zafar et al., 2019, 2017a] etc. It is recognized that there is no unique optimal way to define fairness, leading to trade-offs between different approaches [Zafar et al., 2017b].

Given a fairness criterion, the second component of a fairness strategy requires an algorithmic approach, typically consisting of either 1) pre-processing the data by mapping the training data to a transformed space where the dependencies between sensitive attributes and class labels disappear [Kamiran and Calders, 2012, Dwork et al., 2018]; or 2) postprocessing of a trained prediction model to modify the probability of the decision being positive from an existing classifier to limit unfair discrimination [Kamishima et al., 2012, Hardt et al., 2016]; or 3) “in-process,” where fairness is accounted for during training of a model, e.g., by adding a fairness constraint to the objective function during training. Zemel et al. [2013] proposed to learning a fair representation of the data and classifier parameters by optimizing a non-convex function. Zafar et al. [2017b] further defined a convex function as a measure of (un)fairness, and suggested optimizing accuracy subject to the convex fairness constraints as well as their converse.

A key feature of nearly all existing approaches is that a single set of classifier parameters is estimated, using fairness criteria that encourage fair prediction performance across all groups. This approach has two main limitations: i) fairness is often achieved by compromising accuracy of some groups; ii) the underlying relationship between dependent and independent variables may not be the same across group, and the differences in predictive features may be of interest. In the example of predicting mortality risk for patients with COVID-19, while one would expect some features to have the same association with mortality for both older and younger patients, the associations between mortality and other features may be different between age groups. For instance, overweight and obesity (Body Mass Index [BMI] >25*kg*/*m*^2^) increase the risk for COVID-19 associated mortality, particularly among adults aged < 65 years [Kompaniyets et al., 2021] However, geriatric BMI guidelines are different from younger adults. For older adults, higher BMIs are often associated with greater energy stores and a better nutritional state overall, which is beneficial for patients’ survival outcomes when serious infections are developed. Estimating separate prediction models for each group does not leverage potential similarities between the groups. Moreover, estimating a single prediction model, even with the fairness criteria, will likely result in sub-optimal estimation or prediction performances for one group in order to achieve fair performances with one set of parameters shared across groups. Danaher et al. [2014] proposed the joint graphical lasso method, a technique for jointly estimating multiple models corresponding to distinct but related conditions. Their approach is based upon a penalized log-likelihood approach, which penalizes the differences between parameter estimates across groups. Penalized log-likelihood approaches have often been used by other authors like Yuan and Lin [2007], Friedman et al. [2007b] etc. for similar estimation purposes while minimizing the disparities in estimates across groups. In all such cases, however, prediction performances are not considered.

In this paper, we propose a Joint Fairness Model, a technique for jointly estimating multiple prediction models corresponding to distinct but related groups, to achieve fair prediction performances across groups. The model parameters are estimated by encouraging prediction fairness, while simultaneously ensuring high predictive accuracy irrespective of the heterogeneity across the groups. The rest of this paper is organized as follows. In Section 2, we present the proposed joint fairness model. Section 3 describes the algorithm to find its optimal solution, and discusses hyperparameter selection. In Section 4, we discuss asymptotic consistency of the estimators. We illustrate the performance of our proposal in simulation studies in Section 5; and an application to the motivating example of predicting COVID-19 mortality outcomes for patients of different age groups in Section 6. Section 7 extends the proposed joint fairness model to generalized linear models for other types of outcomes. Finally, we summarize and discuss our findings in Section 8.

## 2 Problem Formulation

For binary outcomes, consider we are given *K* groups of datasets $S^{k}=\left\{\left(X_{i}^{k},\;y_{i}^{k}\right)\in {\mathbb{R}}^{p}\times \left\{0,1\right\}:i=1,\;\cdots ,\;n^{k}\right\}$ with *K* ≥ 2 representing group membership. Assuming that the $n=\sum_{k=1}^{K}\;n^{k}$ observations are independently distributed: $y_{i}^{k}\sim \text{Bernoulli}\left({\text{p}}_{i}^{k}\right)$, ${\hat{y}}_{i}^{k}:{\mathbb{R}}^{p}\to \left\{0,1\right\}$ is the predicted value based on predictor features $X_{i}^{k}$. We focus on the development of the fair prediction approach for the widely-used logistic regression model. The log-likelihood of the logistic model for the data from all groups takes the form

(1) $$\overset{K}{\underset{k=1}{\sum }}l\left(\beta^{k};\;X^{k},\;y^{k}\right)=\overset{K}{\underset{k=1}{\sum }}\overset{n^{k}}{\underset{i=1}{\sum }}\left(y_{i}^{k}X_{i}^{k}\beta^{k}-\text{log}\left(1+\text{exp}\left(X_{i}^{k}\beta^{k}\right)\right)\right).$$

Define $\beta =\left(\beta^{1}\;\ldots \;\beta^{K}\right)\in {\mathbb{R}}^{pK}$. Maximizing the likelihood function (1) with respect to ***β***^*k*^ in each group separately yields the maximum likelihood estimates ${\hat{\beta }}^{k}$ of group *k*, thus making separate predictions *ŷ*^*k*^ per group. If we ignore group memberships, $\hat{\beta }$ can be estimated by maximizing the likelihood function in equation (1) setting all ***β***^*k*^ equal to a single global parameter vector $\hat{\beta }$ and making predictions *ŷ* per individual (irrespective of group) using that parameter vector.

If the *K* datasets correspond to observations collected from *K* distinct but related groups, then one might wish to borrow strength across the *K* groups to estimate ***β*** and predict *ŷ*, rather than estimating parameters ***β***^*k*^ for each group separately, or estimating one set of ***β***^*k*^ for all *k* which can lead to heterogeneous prediction performance across the groups. Therefore, instead of estimating ***β*** by maximizing the likelihood in equation (1), we consider a penalized log-likelihood approach and seek to jointly estimate ***β*** by solving an objective function of $\sum_{k=1}^{K}l\left(\beta^{k};X^{k},\;y^{k}\right)$ in equation (1) subject to constraints on (i) fairness, $P_{\text{F}}\left(\beta ;\;X,\;y,\;\lambda_{\text{F}}\right)$ (ii) parameter similarity, $P_{\text{Sim}}\left(\beta ;\;\lambda_{\text{Sim}}\right)$, and (iii) parameter sparsity, $P_{\text{Sp}}\left(\beta ;\;\lambda_{\text{Sp}}\right)$.

(2) $$\underset{\beta }{\text{minimize}}\;F\left(\beta \right)=-\underset{k}{\sum }\frac{1}{n_{k}}l\left(\beta^{k};\;X^{k},\;y^{k}\right)+P_{\text{F}}\left(\beta ;\;X,\;y,\;\lambda_{\text{F}}\right)+P_{\text{Sim}}\left(\beta ;\;\lambda_{\text{Sim}}\right)+P_{\text{Sp}}\left(\beta ;\;\lambda_{\text{Sp}}\right).$$

We propose choosing a fairness penalty function $P_{\text{F}}\left(\beta ;\;X,\;y,\;\lambda_{\text{F}}\right)$ that encourages each group to have similar predictive performance. In this work, we use equalized odds [Hardt et al., 2016] which encourages each group to have similar false positive rates (FPRs) and false negative rates (FNRs). Thus, we want to minimize the absolute difference between FPR^*j*^ and FPR^*k*^ |*P*(*ŷ* = 1|*G* = *j*, *y* = 0) − *P*(*ŷ* = 1|*G* = *k*, *y* = 0)|, and that between FNR^*j*^ and FNR^*k*^: |*P*(*ŷ* = 0|*G* = *j*, *y* = 1) − *P*(*ŷ* = 0|*G* = *k*, *y* = 1)|.

Under the logistic regression model, $\left|P\left(\hat{y}=1|G=j,\;y=0\right)-P\left(\hat{y}=1|G=k,\;y=0\right)\right|=\left|E\left[\frac{\text{exp}\left(X\beta^{j}\right)}{1+\text{exp}\left(X\beta^{j}\right)}|G=j,\;y=0\right]-E\left[\frac{\text{exp}\left(X\beta^{k}\right)}{1+\text{exp}\left(X\beta^{k}\right)}|G=k,\;y=0\right]\right|$ which is nonconvex due to the nonconvexity of the sigmoid function. We instead minimize the absolute difference of the expected linear components of the two groups $\left|E\left[X\beta^{j}|G=j,\;y=0\right]-E\left[X\beta^{k}|G=k,\;y=0\right]\right|$. The inequality below, which follows from a first order Taylor series approximation of the sigmoid function, guarantees that minimizing the difference of the linear components results in minimizing the difference of the FPRs:

$$\left|E\left[\frac{\text{exp}\left(X\beta^{j}\right)}{1+\text{exp}\left(X\beta^{j}\right)}|G=j,\;y=0\right]-E\left[\frac{\text{exp}\left(X\beta^{k}\right)}{1+\text{exp}\left(X\beta^{k}\right)}|G=k,\;y=0\right]\right|\;\leq \left|E\left[\frac{1}{2}+\frac{X\beta^{j}}{4}|G=j,\;y=0\right]-E\left[\frac{1}{2}+\frac{X\beta^{k}}{4}|G=k,\;y=0\right]\right|.$$

Similar approximation can be used for the absolute difference between FNR^*j*^ and FNR^*k*^. Note that the empirical estimate of the expectation is

$$E\left[X\beta^{k}|G=k,\;y=y\right]=\frac{1}{\left|S_{y}^{k}\right|}\underset{i\in S_{y}^{k}}{\sum }X_{i}\beta^{k},$$

where $S_{y}^{k}=\left\{\left(X_{i},y_{i}\right):G_{i}=k,\;y_{i}=y\right\}$ is a subgroup defined by group *k* and the true response value *y* with *y* ∈ {0, 1}. Thus, our fairness penalty to bridge the between-group gaps in the linear components of FPR^*k*^ and FNR^*k*^ is defined as:

(3) $$P_{\text{F}}\left(\beta ;\;X,\;y,\;\lambda_{\text{F}}\right)=P_{\text{FPR}}\left(\beta ;\;X,\;y,\;\lambda_{\text{F}}\right)+P_{\text{FNR}}\left(\beta ;\;X,\;y,\;\lambda_{\text{F}}\right)\;=\lambda_{\text{F}}\underset{j<k}{\sum }\left|\frac{1}{\left|S_{0}^{j}\right|}\underset{i\in S_{0}^{j}}{\sum }X_{i}\beta^{j}-\frac{1}{\left|S_{0}^{k}\right|}\underset{i\in S_{0}^{k}}{\sum }X_{i}\beta^{k}\right|+\lambda_{\text{F}}\underset{j<k}{\sum }\left|\frac{1}{\left|S_{1}^{j}\right|}\underset{i\in S_{1}^{j}}{\sum }X_{i}\beta^{j}-\frac{1}{\left|S_{1}^{k}\right|}\underset{i\in S_{1}^{k}}{\sum }X_{i}\beta^{k}\right|$$

where the summation $\sum_{j<k}$ represents $\sum_{k=1}^{K}\sum_{j=1}^{k-1}$ for the simplicity.

The similarity penalty $P_{\text{Sim}}\left(\beta ;\;\lambda_{\text{Sim}}\right)$ is chosen to encourage similarity across the *K* estimated parameters. Here we use the generalized fused Lasso penalty [Hoefling, 2010, Danaher et al., 2014, Dondelinger et al., 2018] defined as

(4) $$P_{\text{Sim}}\left(\beta ;\;\lambda_{\text{Sim}}\right)=\lambda_{\text{Sim}}\underset{j<k}{\sum }{\left\|\beta^{j}-\beta^{k}\right\|}_{1}.$$

The sparsity penalty $P_{\text{Sp}}(\beta )$ is chosen to encourage sparse estimates and to avoid ill-defined maximum likelihood estimates when *n*^*k*^ < *p*.

(5) $$P_{\text{Sp}}\left(\beta ;\;\lambda_{\text{Sp}}\right)=\underset{k}{\sum }\lambda_{{\text{Sp}}_{k}}{\left\|\beta^{k}\right\|}_{1}.$$

In the three penalty functions, *λ*_F_, *λ*_Sim_, and *λ*_Sp_ are nonnegative hyperparameters. Here $P_{\text{Sp}}\left(\beta ;\;\;\lambda_{\text{Sp}}\right)$, $P_{\text{F}}\left(\beta ;\;X,\;y,\;\lambda_{\text{F}}\right)$, and $P_{\text{Sim}}\left(\beta ;\;\lambda_{\text{Sim}}\right)$ are convex penalty functions, so that the objective in equation (2) is convex in ***β***. The proposed model jointly estimates ***β*** to achieve fair performances across groups, herein referred to as the Joint Fairness Model (JFM). In contrast, the dominant approach for fair predictions in the current literature is to estimate a single set of ***β*** parameters with constraints on quality of performance metrics across groups [Bechavod and Ligett, 2017].

Penalty functions in (3), (4), and (5) are based on the *L1* norm. They can be flexibly adapted to *L2* penalization or a combination of *L1* and *L2* penalizations. The difference between *L1* and *L2* penalties have been well discussed [Tibshirani, 1996, Zou and Hastie, 2005]. For the fairness penalty, Bechavod and Ligett [2017] showed that there are no remarkable differences in the empirical performances between *L1* and *L2* fairness penalty forms. When we use the *L2* form of the similarity penalty, it penalizes large differences more aggressively so that models have less chance to obtain group-specific estimates. Note that other formats of the similarity penalty can be used in the JFM framework. For example, the group Lasso penalty [Yuan and Lin, 2006] has been shown to encourage similar sparsity patterns across groups [Obozinski et al., 2010, Danaher et al., 2014], while the fused lasso term is more aggressive in encouraging similar ${\hat{\beta }}^{k}$ estimates.

## 3 Accelerated Smoothing Proximal Gradient Algorithm for JFM

In this section, we introduce an Accelerated Smoothing Proximal Gradient (ASPG) Algorithm [Chen et al., 2012] to solve the optimization problem (2) for JFM. The objective function of (2) is convex in ***β*** so that a global optimal solution can be attained. However, conventional proximal gradient-based or coordinate descent approaches (generally used for Lasso-like methods) cannot be directly applied to solve Problem (2) because there is no closed form solution for a proximal operator associated with $P_{\text{FPR}}$ and $P_{\text{FNR}}$.

### 3.1 Nesterov smooth approximation

To overcome the difficulty originating from the non-differentiability of the fairness and similarity penalties, we decouple the terms into a linear combination of the decision variables via the dual norm, then apply the Nesterov smoothing approximation [Nesterov, 2005]. We start with matrix representations of the fairness penalty terms $P_{\text{FPR}}\left(\beta ;\;X,\;y,\;\lambda_{\text{F}}\right)=\lambda_{\text{F}}{\left\|D_{0}\beta \right\|}_{1}$ and $P_{\text{FNR}}\left(\beta ;\;X,\;y,\;\lambda_{\text{F}}\right)=\lambda_{\text{F}}{\left\|D_{1}\beta \right\|}_{1}$, where $D_{y}\in {\mathbb{R}}^{K\left(K-1\right)/2\times pK}$ is defined as below. Similarly, the matrix representation of the similarity penalty $P_{\text{Sim}}\left(\beta ;\;\lambda_{\text{Sim}}\right)=\lambda_{\text{Sim}}\|F\beta {\|}_{1}$ with **F** defined as below.

$${\text{D}}_{y}=\left(\begin{array}{ccccc} {\bar{\text{X}}}_{y}^{1} & -{\bar{\text{X}}}_{y}^{2} & 0 & \cdots & 0\\ & & \vdots & & \\ 0 & {\bar{\text{x}}}_{y}^{2} & -{\bar{\text{X}}}_{y}^{3} & \cdots & 0\\ & & \vdots & & \end{array}\right)\text{ F}=\left(\begin{array}{ccccc} {\text{I}}_{p} & -{\text{I}}_{p} & 0 & \cdots & 0\\ & & \vdots & & \\ 0 & {\text{I}}_{p} & -{\text{I}}_{p} & \cdots & 0\\ & & \vdots & & \end{array}\right)$$

Here, ${\bar{X}}_{y}^{j}=\frac{1}{\left|S_{y}^{j}\right|}\sum_{X^{j}\in S_{y}^{j}}X^{j}$ is the average logit vector for group *j* with outcome *y*, **I**_*p*_ is the *p*-dimensional identity matrix. The single matrix form of the fairness penalty term and the similarity penalty term is therefore defined as:

$$P_{\text{F}}\left(\beta ;\;X,\;y,\;\lambda_{\text{F}}\right)+P_{\text{Sim}}\left(\beta ;\;\;\lambda_{\text{Sim}}\right)={\left\|\left(\begin{array}{c} \lambda_{\text{F}}D_{0}\\ \lambda_{\text{F}}D_{1}\\ \lambda_{\text{Sim}}F \end{array}\right)\beta \right\|}_{1}={\left\|D_{\lambda_{\text{F}},\lambda_{\text{Sim}}}\beta \right\|}_{1}.$$

Thus, the objective function (2) can be written in matrix form:

(6) $$\underset{\beta }{\text{minimize}}\;-\underset{k}{\sum }l\left(\beta^{k};\;X^{k},\;y^{k}\right)+{\left\|D_{\lambda_{\text{F}},\lambda_{\text{Sim}}}\beta \right\|}_{1}+\underset{k}{\sum }\lambda_{{\text{Sp}}_{k}}{\left\|\beta^{k}\right\|}_{1},$$

where the associated proximal operator of ${\left\|{\text{D}}_{\lambda_{\text{F}},\lambda_{\text{Sim}}}\beta \right\|}_{1}$ does not have a closed form solution. We apply the Nesterov smooth approximation to approximate ${\left\|{\text{D}}_{\lambda_{\text{F}},\lambda_{\text{Sim}}}\beta \right\|}_{1}$ by a smooth function *f*_*μ*_(***β***). Since the dual norm of the *L1* norm is the *L*_∞_ norm, we have

$${\left\|{\text{D}}_{\lambda_{\text{F}},\lambda_{\text{Sim}}}\beta \right\|}_{1}=\text{sup}\left\{\alpha^{T}{\text{D}}_{\lambda_{\text{F}},\lambda_{\text{Sim}}}\beta :\|\alpha {\|}_{\infty }\leq 1\right\},$$

and thus, for *μ* > 0, Nesterov smooth approximation of ${\left\|{\text{D}}_{\lambda_{\text{F}},\lambda_{\text{Sim}}}\beta \right\|}_{1}$ is

(7) $$f_{\mu }\left(\beta ;\;\lambda_{\text{F}},\;\lambda_{\text{Sim}}\right)=\text{sup}\left\{\alpha^{T}D_{\lambda_{\text{F}},\lambda_{\text{Sim}}}\beta -\frac{\mu }{2}\|\alpha {\|}_{2}^{2}:\|\alpha {\|}_{\infty }\leq 1\right\}.$$

The following proposition provides the maximum gap between ${\left\|{\text{D}}_{\lambda_{\text{F}},\lambda_{\text{Sim}}}\beta \right\|}_{1}$ and its Nesterov approximation *f*_*μ*_(***β***; *λ*_F_, *λ*_Sim_).

#### Proposition 3.1

For any *μ* > 0, the Nesterov smooth approximation satisfies the following inequalities:

$$0\leq {\left\|D_{\lambda_{\text{F}},\lambda_{\text{Sim}}}\beta \right\|}_{1}-f_{\mu }\left(\beta ;\;\lambda_{\text{F}},\;\lambda_{\text{Sim}}\right)\leq \frac{\mu pK}{2}.$$

Proof: See Supplementary Material S.2.

The proposition implies that we can control the upper bound of the approximation error by manipulating *μ*. We can achieve an arbitrary accuracy *δ* by letting $\mu =\frac{2\delta }{pK}$.

The next proposition dictates that the gradient ∇*f*_*μ*_(***β***; *λ*_F_, *λ*_Sim_) has a simple form and is thus easy to compute.

#### Proposition 3.2

For any *μ* > 0, *f*_*μ*_(***β***; *λ*_*F*_, *λ*_*Sim*_) is smooth and convex with respect to ***β***, whose gradient takes the following form:

(8) $$\nabla f_{\mu }\left(\beta ;\;\lambda_{\text{F}},\;\lambda_{\text{Sim}}\right)={\text{D}}_{\lambda_{\text{F}},\lambda_{\text{Sim}}}^{T}\alpha^{*},$$

where $\alpha^{*}=\text{argmax}\left\{\alpha^{T}D_{\lambda_{\text{F}},\lambda_{\text{sim}}}\beta -\frac{\mu }{2}\|\alpha {\|}_{2}^{2}:\|\alpha {\|}_{\infty }\leq 1\right\}$. Moreover, the gradient is Lipschitz continuous with the Lipschitz constant $L_{\mu }=\mu^{-1}{\left\|D_{\lambda_{\text{F}},\lambda_{\text{Sim}}}\right\|}_{2}^{2}$ where ∥ · ∥_2_ denotes the matrix spectral norm (which is equivalent to the largest singular value of the matrix).

Proof: See Supplementary Material S.3.

#### Computational Remark:

Matrix multiplication $D_{\lambda_{\text{F}}\lambda_{\text{Sim}}}^{T}\alpha^{*}$ requires $O\left(p^{2}K^{3}\right)$ operations, thus making it computationally intensive when *p* is large. However, $D_{\lambda_{\text{F}}\lambda_{\text{Sim}}}^{T}\alpha^{*}$ can be computed efficiently without matrix multiplication. Because of its special structure, its computation can be substituted by a series of scalar multiplications and vector additions. We can reduce the complexity to $O\left(pK^{3}\right)$. Details are provided in Supplementary Material S.1.

The following proposition yields to attain α* in Proposition 3.2, which is essential to compute the gradient ∇*f*_*μ*_(***β***; *λ*_F_, *λ*_Sim_).

#### Proposition 3.3

For any *μ* > 0, we have

$$\alpha^{*}=S_{\infty }\left(\mu^{-1}{\text{D}}_{\lambda_{\text{F}},\lambda_{\text{Sim}}}\beta \right),$$

where *S*_*∞*_(·) is the projection onto the unit *L*_*∞*_ ball, which is defined by

$${\left[S_{\infty }\left(x\right)\right]}_{i}=\{\begin{array}{ll} x_{i} & \text{ if }x_{i}\in \left[-1,\;1\right]\\ 1 & \text{ if }x_{i}\in \left(1,\;\infty \right)\\ -1 & \text{ if }x_{i}\in \left(-\infty ,\;-1\right) \end{array}.$$

Proof: See Supplementary Material S.4.

#### Computational Remark:

The matrix multiplication $D_{\lambda_{\text{F}},\lambda_{\text{Sim}}}\beta$ is computationally expensive as well. It requires $O\left(p^{2}K^{3}\right)$ operations, however, we can simplify it to $O\left(pK^{2}\right)$ by performing a series of vector subtractions. The details are presented in Supplementary Material S.1.

### 3.2 Accelerated Smoothing Proximal Gradient Algorithm

With ${\left\|{\text{D}}_{\lambda_{\text{F}},\lambda_{\text{Sim}}}\beta \right\|}_{1}$ substituted by the Nesterov smooth approximation *f*_*μ*_(***β***; *λ*_F_, *λ*_Sim_), Problem (6) becomes

(9) $$\underset{\beta }{\text{minimize}}\;\tilde{F}\left(\beta \right)=-\underset{k}{\sum }l\left(\beta^{k};\;X^{k},\;y^{k}\right)+f_{\mu }\left(\beta ;\;\lambda_{\text{F}},\;\lambda_{\text{Sim}}\right)+\underset{k}{\sum }\lambda_{{\text{Sp}}_{k}}{\left\|\beta^{k}\right\|}_{1},$$

whose first two terms are convex smooth functions. Although the sparsity penalty term $\sum_{k}\lambda_{{\text{Sp}}_{k}}{\left\|\beta^{k}\right\|}_{1}$ is non-differentiable, it can be managed through the proximal gradient method using the soft-thresholding operator S with a closed form solution [Friedman et al., 2007a].

Algorithm 1 presents the proposed ASPG algorithm, starting from parameter initialization, to gradient descent iterations with proximal and momentum steps, until convergence. The gradient descent step tries to improve the current solution *γ*^(*t*−1)^ by using the gradients ∇*ℓ* of the log-likelihood and ∇*f*_*μ*_ of function (8). Subsequently, it performs a proximal step for the sparsity penalty. Finally, a momentum-based update is performed to accelerate the convergence. Specifically, we adopted the momentum coefficients in the fast iterative shrinkage thresholding algorithm [Beck and Teboulle, 2009].

Although Algorithm 1 minimizes the Nesterov smooth approximation $\tilde{F}\left(\beta \right)$ instead of the original objective function *F*(***β***) in equation (2), it can be proven that the solution is sufficiently close to the optimal solution of equation (2). We first present a lemma demonstrating a convergence property of the algorithm.

#### Lemma 3.1

Let {***β***^(*t*)^ : *t* = 1, 2, ···} be a sequence generated by Algorithm 1. Then for any *t* ≥ 1,

$$\tilde{F}\left(\beta^{\left(t\right)}\right)-\tilde{F}\left(\beta^{*}\right)\leq \frac{2L{\left\|\beta^{\left(0\right)}-\beta^{*}\right\|}_{2}^{2}}{t^{2}},$$

where β* is a global minimizer of Problem (9).

Proof: Proof of this theorem is analogous to the proof of Theorem 4.4 in Beck and Teboulle [2009] because $-\sum_{k}l\left(\beta^{\left(k\right)};\;X^{k},\;y^{k}\right)+f_{\mu }\left(\beta ;\;\lambda_{\text{F}},\;\lambda_{\text{Sim}}\right)$ is a convex differentiable function and it has Lipschitz continuous gradient with Lipschitz constant

$$L=\frac{1}{4}\text{max}\left\{\lambda_{\text{max}}\left(X^{kT}X^{k}\right):k=1,\;\cdots ,\;K\right\}+\mu^{-1}{\left\|D_{\lambda_{\text{F}},\lambda_{\text{Sim}}}\right\|}_{2}^{2}>0,$$

where *λ*_max_(**A**) denotes the largest eigenvalue of **A**.


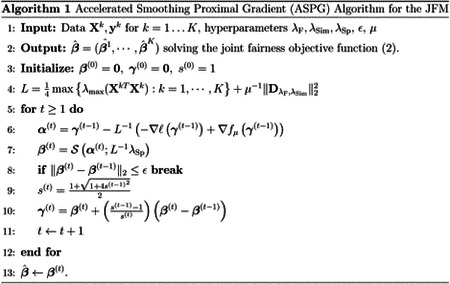


Based on the lemma, we establish a theorem that shows the solution provided by Algorithm 1 can be arbitrarily close to the global optimum of Problem (2).

#### Theorem 3.1

Let {***β***^(*t*)^ : *t* = 1, 2, ···} be a sequence generated by Algorithm 1. Then for any *t* ≥ 1,

$$F\left(\beta^{\left(t\right)}\right)-F\left(\beta^{**}\right)\leq \frac{\mu pK}{2}+\frac{2L{\left\|\beta^{\left(0\right)}-\beta^{*}\right\|}_{2}^{2}}{t^{2}},$$

where β* and β** are global minimizers of Problem (9) and Problem (2), respectively, and *L* is the Lipschitz constant of $\tilde{F}$ presented in Lemma 3.1.

Proof: We can easily verify the inequality by applying Proposition 3.1 and Lemma 3.1, and using $\tilde{F}\left(\beta^{*}\right)\leq \tilde{F}\left(\beta^{**}\right)$ as below:

$$F\left(\beta^{\left(t\right)}\right)-F\left(\beta^{**}\right)=\left(F\left(\beta^{\left(t\right)}\right)-\tilde{F}\left(\beta^{\left(t\right)}\right)\right)+\left(\tilde{F}\left(\beta^{\left(t\right)}\right)-\tilde{F}\left(\beta^{*}\right)\right)+\left(\tilde{F}\left(\beta^{*}\right)-F\left(\beta^{**}\right)\right)\;\leq \frac{\mu pK}{2}+\frac{2L{\left\|\beta^{0}-\beta^{*}\right\|}_{2}^{2}}{t^{2}}+0.$$

Given the desired accuracy *δ* > 0 for the approximation, we set $\mu =\frac{2\delta }{pK}$. Then, we have $F\left(\beta^{\left(t\right)}\right)-F\left(\beta^{**}\right)\leq \delta +\frac{2L{\left\|\beta^{0}-\beta^{*}\right\|}_{2}^{2}}{t^{2}}$. This inequality implies that the accuracy of Algorithm 1 both depends on the number of iterations *t* and the accuracy *δ* > 0 for the approximation. Based on the theorem, we present the rate of convergence of the algorithm in the following proposition.

#### Proposition 3.4

Given a desired accuracy *ε* > 0, rate of convergence of Algorithm 1 is $O\left(\sqrt{\frac{pK}{\delta \left(\varepsilon -\delta \right)}}\right)$. Note that *δ* > 0 must be smaller than *ε*.

Proof: See Supplementary Material S.5.

#### Proposition 3.5

Time complexity of a single iteration of Algorithm 1 is $O\left(\left(n+K^{2}\right)pK\right)$.

Proof: Computing the gradient ∇Σ_*k*_ *ℓ*(***β***^*k*^) of the sum of the log-likelihood functions requires O(npK). Computing ∇*f*_*μ*_(***β***; *λ*_F_, *λ*_Sim_) requires $O\left(pK^{3}\right)$. Thus, the gradient step requires $O\left(\left(n+K^{2}\right)pK\right)$ operations. The proximal step and momentum step both require O(pK), which are dominated by the complexity of the gradient step. Therefore, a single iteration of Algorithm 1 requires $O\left(\left(n+K^{2}\right)pK\right)$ operations.

## 4 Asymptotic properties of the JFM estimates

We now present the key asymptotic results for the JFM parameter estimates $\hat{\beta }$ for each group by solving objective function (2) of a logistic regression for a binary outcome when *K* = 2. We assume *p* remains constant and *n* increases to infinity. Consider the following assumptions

### Assumption 1

$I\left(\beta^{k}\right)/n^{k}\to C^{k}$, where **C**^*k*^ is a positive definite *p* × *p* matrix, for *k* = 1 and 2, where $I\left(\beta^{k}\right)$ is the information matrix of size *p*×*p*. For simplicity, we assume there are no intercept terms in ***β***^*k*^.

### Assumption 2.

As $n=\underset{k=1,2}{\text{min}}\;n^{k}\to \infty$, ${\text{max}}_{{\hat{\beta }}^{k}}{\left\|\left(I{\left({\hat{\beta }}^{k}\right)}^{-\frac{1}{2}}\right)I\left(\beta^{k}\right){\left(I{\left({\hat{\beta }}^{k}\right)}^{-\frac{1}{2}}\right)}^{T}-I_{p}\right\|}_{2}\to 0$ where $I\left({\hat{\beta }}^{k}\right)$ is the empirical information matrix, and **I**_*p*_ is a *p* × *p* identity matrix.

The following theorem proves $\sqrt{n}$-consistency for the estimators, complying with the fairness and similarity constraints between the two groups as well as the sparsity constraint.

### Theorem 4.1

Let ${\hat{\beta }}^{k}$ for *k* = 1 and 2, minimize the loss function (2). If $\lambda_{\text{F}}^{\left(n\right)}/\sqrt{n}\to \lambda_{\text{F}}^{\left(0\right)}\geq 0$, $\lambda_{\text{Sim}}^{\left(n\right)}/\sqrt{n}\to \lambda_{\text{Sim}}^{\left(0\right)}\geq 0$, and $\lambda_{\text{Sp}}^{\left(n\right)}/\sqrt{n}\to \lambda_{\text{Sp}}^{\left(0\right)}\geq 0$, then under the assumptions 1 and 2

(10) $$\sqrt{n}\left({\hat{\beta }}^{k}-\beta^{k}\right)\to {\hat{u}}^{k}$$

where $\left\{{\hat{u}}^{1},\;{\hat{u}}^{2}\right\}=\text{argmin}\left(V\right)$, for $u^{k}=\left(u_{1}^{k},\;\ldots ,\;u_{p}^{k}\right)\in {\mathbb{R}}^{p}$,

$$V\left(u^{1},\;u^{2}\right)=u^{1T}W^{1}+u^{2T}W^{2}+\frac{1}{2}u^{1T}C^{1}u^{1}+\frac{1}{2}u^{2T}C^{2}u^{2}+\;\lambda_{\text{F}}^{\left(0\right)}[\left({\bar{\text{X}}}_{0}^{1}u^{1}-{\bar{X}}_{0}^{2}u^{2}\right)\text{sign}\left({\bar{X}}_{0}^{1}\beta^{1}-{\bar{X}}_{0}^{2}\beta^{2}\right)I\left({\bar{X}}_{0}^{1}\beta^{1}\neq {\bar{X}}_{0}^{2}\beta^{2}\right)+\left|{\bar{X}}_{0}^{1}u^{1}-{\bar{X}}_{0}^{2}u^{2}\right|I\left({\bar{X}}_{0}^{1}\beta^{1}={\bar{X}}_{0}^{2}\beta^{2}\right)+\;\left({\bar{\text{X}}}_{1}^{1}u^{1}-{\bar{X}}_{1}^{2}u^{2}\right)\text{sign}\left({\bar{X}}_{1}^{1}\beta^{1}-{\bar{X}}_{1}^{2}\beta^{2}\right)I\left({\bar{X}}_{1}^{1}\beta^{1}\neq {\bar{X}}_{1}^{2}\beta^{2}\right)+\left|{\bar{X}}_{1}^{1}u^{1}-{\bar{X}}_{1}^{2}u^{2}\right|I\left({\bar{X}}_{1}^{1}\beta^{1}={\bar{X}}_{1}^{2}\beta^{2}\right)]+\;\lambda_{\text{Sp}}^{\left(0\right)}\sum_{k=1}^{2}\sum_{j=1}^{p}\left\{u_{j}^{k}\text{sign}\left(\beta_{j}^{k}\right)I\left(\beta_{j}^{k}\neq 0\right)+\left|u_{j}^{k}\right|I\left(\beta_{j}^{k}=0\right)\right\}+\;\lambda_{\text{Sim}}^{\left(0\right)}\sum_{j=1}^{p}\left\{\left(u_{j}^{1}-u_{j}^{2}\right)\text{sign}\left(\beta_{j}^{1}-\beta_{j}^{2}\right)I\left(\beta_{j}^{1}\neq \beta_{j}^{2}\right)+\left|u_{j}^{1}-u_{j}^{2}\right|I\left(\beta_{j}^{1}=\beta_{j}^{2}\right)\right\}$$

Here $W^{k}\sim N_{p}\left(0,\;C^{k}\right)$, where $C^{k}={\text{lim}}_{n\to \infty }\frac{1}{n}\sum_{i=1}^{n}X_{i}^{k}X_{i}^{kT}$, and $\frac{1}{\left|S_{y}^{k}\right|}\underset{i\in S_{y}^{k}}{\sum }X_{i}={\bar{X}}_{y}^{k}$, for *y* = 0, 1 and *k* = 1, 2.

Proof: See Supplementary Material S.6.

## 5 Simulation Study

We performed a series of simulations to evaluate the proposed JFM, and compared it with the approaches of a group-separate individual logistic regression model, a group-ignorant vanilla logistic regression model, and a Single Fairness Model (SFM) proposed by Bechavod and Ligett [2017]. In the context of logistic regression, such an SFM minimizes the following objective function. We also established ASPG for SFM (see S.8.)

$$\underset{\beta }{\text{minimize}}\;-\underset{k}{\sum }l\left(\beta ;\;X_{k},\;y_{k}\right)+\lambda_{\text{F}}\{\underset{j<k}{\sum }\left|\frac{\sum_{X^{j}\in S_{0}^{j}}X^{j}\beta }{\left|S_{0}^{j}\right|}-\frac{\sum_{X^{k}\in S_{0}^{k}}X^{k}\beta }{\left|S_{0}^{k}\right|}\right|\;+\underset{j<k}{\sum }\left|\frac{\sum_{X^{j}\in S_{1}^{j}}X^{j}\beta }{\left|S_{1}^{j}\right|}-\frac{\sum_{X^{k}\in S_{1}^{k}}X^{k}\beta }{\left|S_{1}^{k}\right|}\right|\}+\lambda_{\text{Sp}}\|\beta {\|}_{1}.$$

When applying the group-separate model, regression coefficients were estimated for each group separately with *L1* penalty. The group-ignorant model estimated one logistic regression with group membership as an additional covariate with an *L1* penalty.

### 5.1 Simulation Setup

We consider a two-group problem (*K* = 2) for simplicity with group 1 as the over-represented group and group 2 as the under-represented group with respect to the sample sizes. The training samples were simulated as follows. The predictor matrix **X**^*k*^ was independently generated from a standard normal distribution. The binary outcome $y_{i}^{k}$ was then simulated from Bernoulli$\left(\pi_{i}\left(x_{i}^{k}\right)\right)$, where $\pi_{i}\left(x_{i}^{k}\right)=\frac{\text{exp}\left(x_{i}^{k}\beta^{k}\right)}{1+\text{exp}\left(x_{i}^{k}\beta^{k}\right)}$. Out of the total number of features, 40% in each group had non-zero coefficients (*β*’s). The non-zero coefficients were each set to the value 3. The simulations were conducted under four scenarios to investigate performances at various levels of shared parameters, sample sizes and dimensionalities.

- In Scenario 1, the shared features between the two groups ranged from 0% to 100% of features with non-zero coefficients. The intercepts were selected so that the baseline event prevalence were at 10% for each group. The sample sizes were set at 500 and 200 for group 1 and 2 respectively. The number of features were set to *p* = 100.
- In Scenario 2, the baseline prevalence of the under-represented group (group 2) ranged from 10% to 50%. The baseline event prevalence of the over-represented group (group 1) was fixed at 50%. Half of the features with non-zero coefficients were shared between the groups, while the other half of the features were group-specific. The sample sizes were set at 500 and 200 for group 1 and 2 respectively. The number of features was set to *p* = 100.
- In Scenario 3, the sample size of the under-represented group (group 2) ranged from 50 to 300 while the sample size of group 1 was fixed at 500. The number of features were set to *p* = 100. Half of the features with non-zero coefficients were shared between the groups.
- In Scenario 4, the number of features *p* ranged from 50 to 2,000 in order to investigate model performance in high-dimensional settings. Sample sizes were 500 and 200 for group 1 and 2 respectively. For each value of *p*, 40 features had non-zero coefficients, with half of the non-zero features shared between the two groups.

We evaluated the methods on independent testing datasets with large sample sizes (*n* = 1000 for both groups) under the same simulation setups. The Area under the Receiver Operating Characteristic curve (AUC) was used to assess the predictive ability of each model. Prediction unfairness was assessed by the group difference in AUCs. Medians and interquartile ranges (IQRs) of the assessment metrics were generated from 20 replicates for each experiment. Predictive performances and their unfairness in terms of FPR and FNR were calculated with cutoff of the predicted probability at 0.5 and presented in Supplementary Material S.10. We further presented additional simulation scenarios in Supplementary Material S.11.

### 5.2 Choice of the Evaluation Metrics in Selecting Hyperparameters in Cross-validations

The group-ignorant model, group-separate model, SFM, and JFM contain 1, *K*, 2, and *K* + 2 hyperparameters respectively. For every method, 5-fold cross-validation on the training dataset was used to determine the hyperparameters. For the vanilla models (group-separate and group-ignorant), the lasso penalty term was selected by optimizing cross-validation AUCs. For the fairness-aware models, we compared a series of evaluation metrics for selecting the hyperparameters in cross-validations, including group average of AUCs/accuracies (arithmetic mean, geometric mean, and harmonic mean), overall AUCs/accuracies on all samples ignoring group memberships, and the group average of AUCs/accuracies subtracting the disparity of AUCs/accuracies (absolute differences and squared differences) in Supplementary Materials S.9. The harmonic mean of group-wise AUCs in cross-validations selected the hyperparameters generating the most robust AUCs and parities in the test datasets, therefore was used in the following simulations results. Besides

### 5.3 Simulation Results

**Figure 1:**
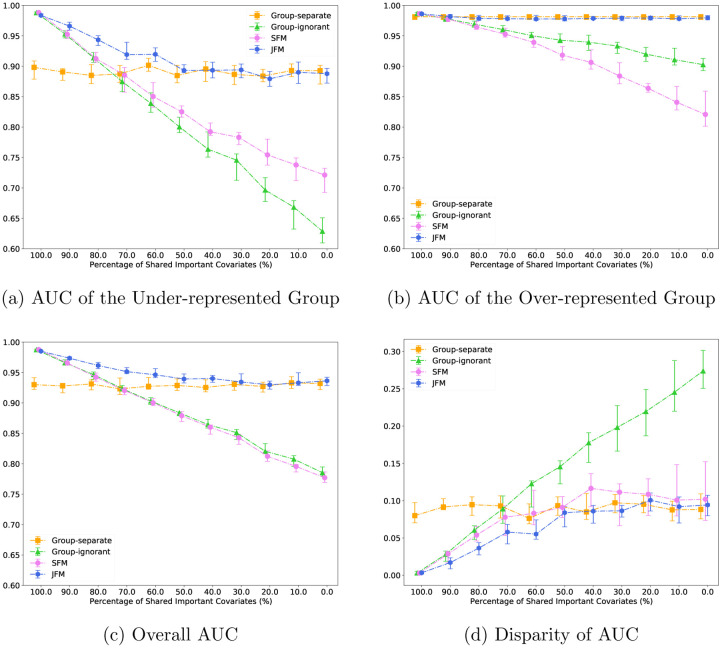
Experimental Results for Scenario 1

For Scenario 1, Figure 1(a) displays the estimated AUC for the under-represented group against the proportion of shared features in the two groups. The AUCs of the under-represented group from the JFM, SFM, and group-ignorant models improved as the proportion of shared features increased. The SFM and group-ignorant models were highly sensitive to the percentage of shared nonzero features as they both estimate a single set of parameters for both groups. In contrast, JFM showed consistently higher AUC than the other three methods. When the proportion of shared features is high, JFM estimated higher AUCs and smaller variances than those from the group-separate model. JFM’s performance was similar to those of the SFM and the group-ignorant model. When the proportion of shared features is low, JFM estimated higher AUCs than the SFM and the group-ignorant model, and showed similar AUC to the group-separate model. Figure 1(b) displays the estimated AUC for the majority group against the proportion of shared features in the two groups. JFM was robust in achieving comparable AUC to that from the group-separate model. The SFM and group-ignorant models were highly sensitive to the percentage of shared features for the majority group with lower AUCs when the proportion of shared parameters is low. Figure 1(c) displays the estimated overall AUCs, and Figure 1(d) displays the group disparity of AUCs from the four approaches. Together, these figures demonstrate that the JFM achieves fair prediction performances robustly across the range of varying proportions of shared features between groups, by training the classifiers jointly with a flexible parameterization. Figure S.5(a) through Figure S.5(d) compares the average of TPR and TNR and disparity in TPR and TNR differences of the four methods. The patterns are similar to those found using AUCs.

**Figure 2:**
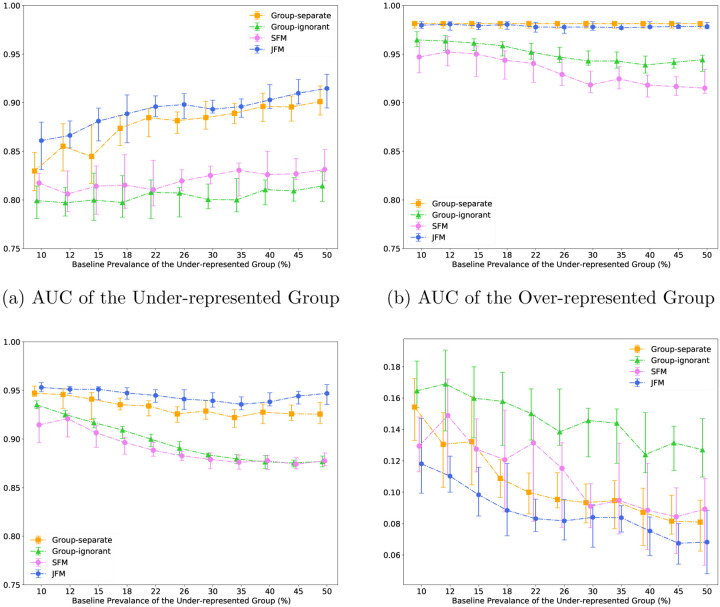
Experimental Results for Scenario 2

Figure 2 displays the performance of the four methods when varying the baseline event prevalence of the under-represented group while holding the prevalence of the majority group fixed. In Figure 2(a), the JFM showed consistently higher AUCs for the underrepresented group than those from all the other models. The AUCs estimated from the group-separate method showed higher variance when the prevalence is rare. Figure 2(b) indicates that the AUC of the over-represented group was not impacted for the JFM and group-separate methods, remaining consistently higher than those from the SFM and the group-ignorant models. As seen in Figure 2(c) and 2(d), the JFM achieves overall satisfactory AUCs and parity between groups with varying sample sizes of the under-represented group. Figure S.6(a) through Figure S.6(d) compares the average of TPR and TNR and disparity in TPR and TNR differences of the four methods.

**Figure 3:**
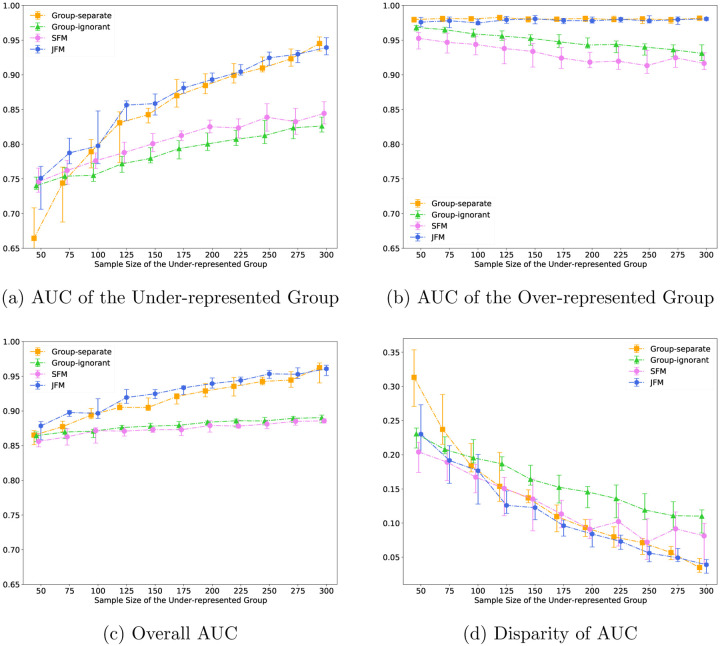
Experimental Results for Scenario 3

Figure 3 displays the performance of the four methods against the sample size of the under-represented group with other settings fixed. In Figure 3(a), the AUCs of the under-represented group from all models were improved as its sample size increased. The JFM showed consistently higher AUCs and smaller variances than those from all the other models. JFM outperforms the other models the most when the minority group’s sample size is small, showing the benefits of borrowing information between groups in situations with unbalanced sample sizes. Figure 3(b) illustrates that the AUC of majority group was not impacted for the JFM and group-separate methods. However, the AUC of the majority group decreased as sample size of the under-represented group increased for the SFM and the group-ignorant models. This decrease highlights an undesirable performance from these two methods, namely, compromising accuracy by estimating a single set of classifier parameters. Figure 3(c) and 3(d) illustrates that the JFM achieves overall satisfactory AUCs and parity between groups across varying sample sizes of the under-represented group. Figure S.7 compares the average of TPR and TNR and disparity of TPR and TNR of the four methods.

**Figure 4:**
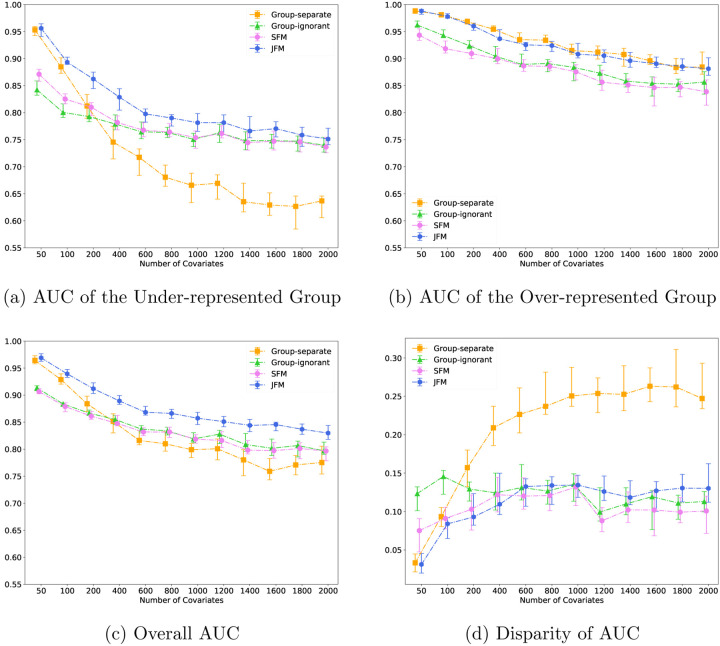
Experimental Results for Scenario 4

Figure 4 displays the performance of the four methods while varying the number of features from 200 to 2000, and holding the number of associated features constant at 40. It demonstrates that the JFM method in going from low dimensional to high dimensional settings can maintain overall satisfactory prediction performances and parity between groups. Supplementary Figure S.12 displays the performance of the four methods while varying the number of features from 200 to 2000, and setting the number of associated features to a fixed proportion of the total number of features. The resultant patterns are similar to Figure 4.

We investigated the empirical computational complexity of JFM with the increasing number of features and sample sizes in the Supplementary Materials. Figure S.1 shows that the JFM computation time is approximately $O\left(p^{1.5}\right)$ and O(n). Details are presented in Section S.7.

## 6. COVID-19 Risk Prediction Case Study

We applied the JFM, in comparison with other methods, to predict mortality related to COVID-19 from patients’ routine ambulatory encounters and laboratory records prior to COVID-19 infection, with the goal of better stratifying patient risk for clinical management. We used a retrospective EHR dataset of 11,594 patients of age 50+ with laboratory-confirmed COVID-19 at New York University Langone Health (NYULH) from March 2020 to February 2021. Among the 11,594 patients, 1,242 (10.7%) died of COVID-19. The patients were divided into four groups by their age at the time of COVID-19 diagnosis: 50–64, 65–74, 75–84, and 85+ with 5, 905 (50.9%), 2, 946 (25.4%), 1, 814 (15.6%), and 929 (8.0%) patients, respectively. The observed mortality rates were 4.44%, 11.17%, 18.96% and 33.05%, respectively. Candidate features (*p* = 82) included demographic variables, such as age, sex, race/ethnicity, smoking status, body mass index (BMI); common chronic disease history such as diabetes, dementia, chronic kidney diseases (CKD); Myocardial Infarction (MI) & Atrial Fibrillation (AF); and routinely collected laboratory markers, such as lipid panels, blood panels, albumin, creatinine, aspartate aminotransferase (AST) etc. obtained from patients routine ambulatory histories before their COVID-19 infections. To build the prediction models, we randomly split the dataset into training (*n* = 8, 115, 70%) and testing (*n* = 3, 479, 30%) sets. We first standardized all features to zero-mean and unit variance. Five-fold cross-validation was conducted on the training set to determine the optimal hyperparameters for each model. Hyperparameters for the group-separate and group-ignorant models were selected to maximize the groupwise AUCs and the overall AUC, respectively, while those for the SFM and JFM were determined to maximize the harmonic mean of groupwise AUCs. Subsequently, we trained the final models with the optimal hyperparameters using the entire training set and applied the final models to the testing dataset to demonstrate their predictive performance. We repeated the training/testing split 10 times and averaged the performances across the 10 times. Table 1 presents the AUCs and the averages of TPR and TNR of the four methods for each age group. The JFM performed better across all age groups than the separate model did, demonstrating that joint modeling yields higher efficiency. Compared with the group ignorant model, the JFM performed better in the three older age groups, with comparable AUC for the 50–64 age group, which resulted in smaller disparities in prediction performance overall. This phenomenon supports the observed pattern in simulation studies that the JFM reduced disparities in prediction performances without impacting those from the majority groups. In contrast, the SFM tended to reduce prediction disparities by lowering the performances for the majority groups.

**Figure 5:**
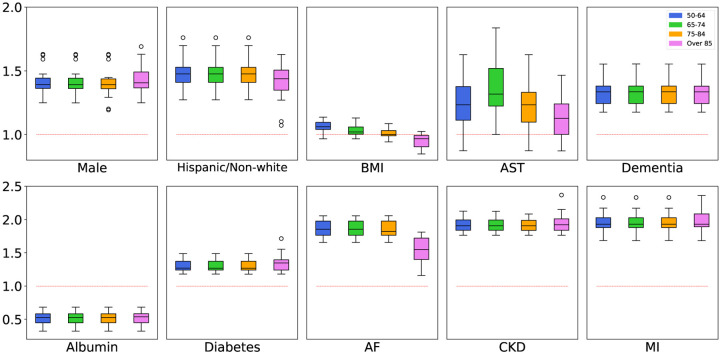
Estimated Odds Ratios for COVID-19 Dataset

Figure 5 presents the boxplots of odds ratios (ORs) of selected demographic and clinical features estimated by the JFM. These results support the hypothesis that some features have common associations between groups, and some have group-specific ORs. For example, the decreasing OR estimates of BMI along age-groups confirmed the prior hypothesis that the association between BMI and COVID-19 mortality is heterogeneous between agegroups. In JFM estimates, BMI is positively associated with higher risks of COVID-19 mortality for patients younger than 75, but with smaller and even reversed ORs in the oldest age groups. For older adults, higher BMIs are often associated with greater energy stores and a better nutritional state overall, which is beneficial for patients’ survival outcomes when infected by COVID-19. The proportion of underweight patients (BMI<18) increased from 0.6% in the age group 50–64 to 5.5% in the age group 85+. The underweight status, often a proxy of frailty, has been repeatedly reported as a strong risk factor of COVID-19-induced multiorgan failure and mortality in older patients [Tehrani et al., 2021]. On the other hand, the JFM can improve efficiencies for covariates with rare prevalence in a subgroup. For instance, dementia has been reported as a risk factor with COVID-19 mortality. In the group-separate model, dementia was insignificant in patients aged 50–64, mainly due to its low prevalence in this group (0.6%). In contrast, dementia was significantly associated with mortality in all age groups with similar ORs in the JFM estimates.

## 7 JFM for Generalized Linear Models

The proposed JFM framework in (2) can be extended to Generalized Linear Models (GLMs) when the response variable **y**^*k*^ is obtained from an exponential family. We can choose a generalized fairness penalty function to encourage each group to have similar linear components.

$$P_{\text{F}}\left(\beta ;\;X,\;y,\;\lambda_{\text{F}}\right)=\lambda_{\text{F}}\underset{j<k}{\sum }E_{y}\left[\left|E\left[X\beta^{j}|G=j,\;y=y\right]-E\left[X\beta^{k}|G=k,\;y=y\right]\right|\right],$$

The proposed accelerated smoothing proximal gradient method can also be extended to solve the generalized JFMs.

## 8 Conclusions and Discussion

In this study we introduced a new method, the joint fairness model, for jointly estimating sparse parameters on the basis of observations drawn from distinct but related groups with the goal of achieving fair performances across groups. We employ an efficient accelerated smoothing proximal gradient algorithm to solve the joint fair objective function, which has convex penalty functions. Our algorithm is tractable on high-dimensional datasets (thousands of features on thousands of samples.) Further, we presented the asymptotic distributions of parameter estimates ${\hat{\beta }}^{k}$ and provided a framework to perform hypothesis testing of the overall ***β*** or the individual elements of *β*_*j*_. Our JFM predictions outperform competing approaches, including group separate models, group ignorant models and single fairness models, on a range of simulated scenarios.

We note that the JFM’s reliance on separate hyperparameters (*K*+2 hyperparameters) that control sparsity, fairness and similarity can be viewed as a strength rather than a drawback because one can vary separately the amount of similarity, sparsity and fairness to enforce in the group specific estimates. In situations with many groups, further assumptions can be made to reduce the number of sparsity hyperparameters (i.e. $\lambda_{{\text{Sp}}_{k}}=c_{k}\lambda_{\text{Sp}}$). Possible choices of *c*_*k*_ include $\frac{1}{\sqrt{n^{k}}}$ so that sparsity is inversely proportional to the number of samples, and 1 for the simplicity.

As an exception of nearly all existing fairness-aware prediction approaches estimating a single set of classifier parameters, recent studies have proposed to use multi-task learning (MTL) to improve algorithm fairness [Oneto et al., 2019]. However, most MTL researches have focused on joint architecture, optimization, and task relationship learning, which is a different emphasis from the proposed JFM approach to improve risk prediction performance for under-represented populations.

Moving forward, the proposed JFM framework can be extended for time-to-event outcomes by putting similar constraints. It can also be extended to non-linear models by adding a suitable fairness penalty term to the objective function. Given the increasing ability to subclassify diseases according to their molecular features and the recognition that substantial heterogeneity exists in many molecular subtypes, most diseases will be eventually classified into a collection of multiple subtypes with unbalanced sample sizes. Therefore, the proposed JFM has wide application potential to improve prediction efficiencies and reduce subgroup prediction disparities beyond applications addressing gender, race/ethnicity and age disparities.

A Python package implementing the JFM will be made available at https://github.com/hyungrok-do/joint-fairness-model.

## Supplementary Material

1

## Figures and Tables

**Table 1:** Predictive Performance on COVID-19 Case Study

Models	AUCs				Average of TPR and TNR			
	50–64	65–74	75–84	Over 85	50–64	65–74	75–84	Over 85
Group-separate	0.838	0.773	0.709	0.649	0.780	0.722	0.669	0.632
Group-ignorant	**0.855**	0.786	0.735	0.659	**0.803**	**0.731**	0.687	0.639
SFM	0.847	0.774	0.728	0.660	0.791	0.724	0.688	0.640
JFM	0.852	**0.791**	**0.736**	**0.672**	0.794	**0.731**	**0.690**	**0.659**
