# Supplementary material for: Joint Fairness Model with Applications to Risk Predictions for Under-represented Populations
Source: ArXiv. 2021 May 10:arXiv:2105.04648v2. Preprint. [Version 2] (PMC8132236)
Supplement: Supplementary file 1 [file NIHPP2105.04648V2-supplement-1.pdf]

# SUPPLEMENTARY MATERIAL

## S.1 Computational Remark

Although $\mathbf{D}^T_{\lambda_{\mathrm{F}},\lambda_{\mathrm{Sim}}}\boldsymbol{\alpha}^*$ and $\mathbf{D}_{\lambda_{\mathrm{F}},\lambda_{\mathrm{Sim}}}\boldsymbol{\beta}$ in Proposition 3.2 and 3.3 seem computationally expensive due to the high-dimensionality of $\mathbf{D}_{\lambda_{\mathrm{F}},\lambda_{\mathrm{Sim}}} \in \mathbb{R}^{(p+1)K(K-1)\times pK}$, we can reduce the complexity because of their structure.

For $\mathbf{D}^T_{\lambda_{\mathrm{F}},\lambda_{\mathrm{Sim}}}\boldsymbol{\alpha}^*$, we have

$$\mathbf{D}^T_{\lambda_{\mathrm{F}},\lambda_{\mathrm{Sim}}}\boldsymbol{\alpha}^* = \lambda_{\mathrm{F}}\mathbf{D}_0\alpha_1^* + \lambda_{\mathrm{F}}\mathbf{D}_1\alpha_2^* + \lambda_{\mathrm{Sim}}\mathbf{A}^*, \tag{S.1}$$

where

$$\mathbf{A}^* = \begin{pmatrix} \boldsymbol{\alpha}^*_{3+} & \boldsymbol{\alpha}^*_{3+} & \boldsymbol{\alpha}^*_{3+} & \cdots & \mathbf{0} \\ -\boldsymbol{\alpha}^*_{3+} & \mathbf{0} & \mathbf{0} & \cdots & \mathbf{0} \\ \mathbf{0} & -\boldsymbol{\alpha}^*_{3+} & \mathbf{0} & \cdots & \mathbf{0} \\ & & \vdots & & \\ \mathbf{0} & \mathbf{0} & \mathbf{0} & \cdots & \boldsymbol{\alpha}^*_{3+} \\ \mathbf{0} & \mathbf{0} & \mathbf{0} & \cdots & -\boldsymbol{\alpha}^*_{3+} \end{pmatrix},$$

and $\boldsymbol{\alpha}^*_{3+} = (\alpha_3^*, \cdots, \alpha_{pK}^*)$ is sub-vector of $\boldsymbol{\alpha}^*$ that obtained by removing first two elements from it. (S.1) requires scalar-matrix multiplication and matrix addition and thus its computational complexity is $\mathcal{O}(pK^3)$, which is lower than $\mathcal{O}(p^2K^3)$ of the matrix multiplication.

On the other hand, we have

$$\mathbf{D}_{\lambda_{\mathrm{F}},\lambda_{\mathrm{Sim}}}\boldsymbol{\beta} = \begin{pmatrix} \lambda_{\mathrm{F}}\mathbf{D}_0\boldsymbol{\beta} \\ \lambda_{\mathrm{F}}\mathbf{D}_1\boldsymbol{\beta} \\ \lambda_{\mathrm{Sim}}\mathbf{F}\boldsymbol{\beta} \end{pmatrix}. \tag{S.2}$$

Here, $\mathbf{F}$ is a sparse matrix consists of identity matrices and thus $\mathbf{F}\boldsymbol{\beta}$ can be computed

without matrix multiplication by

$$\mathbf{F}\boldsymbol{\beta} = \begin{pmatrix} \boldsymbol{\beta}^1 - \boldsymbol{\beta}^2 \\ \vdots \\ \boldsymbol{\beta}^{K-1} - \boldsymbol{\beta}^K \end{pmatrix}. \tag{S.3}$$

Its complexity is $\mathcal{O}(pK^2)$ which is lower than $\mathcal{O}(p^2K^3)$, the complexity of the standard matrix multiplication for $\mathbf{F}\boldsymbol{\beta}$. Since (S.3) only requires a series of vector subtraction operations, it is more efficient than multiplying large matrices. Note that the complexity of (S.2) is also $\mathcal{O}(pK^2)$ because $\mathbf{D}_0\boldsymbol{\beta}$ and $\mathbf{D}_1\boldsymbol{\beta}$ both require $\mathcal{O}(pK)$ computations.

## S.2 Proof of Proposition 3.1

Note that the proof of Proposition 3.1 to Proposition 3.3 are based on the work of Chen et al. [2012]. The left-hand side of the inequalities is trivial by definition. For the right-hand side, we have

$$\|\mathbf{D}_{\lambda_{\mathrm{F}},\lambda_{\mathrm{Sim}}}\boldsymbol{\beta}\|_1 - f_\mu(\boldsymbol{\beta};\lambda_{\mathrm{F}},\lambda_{\mathrm{Sim}}) \leq \frac{\mu}{2}\|\boldsymbol{\alpha}\|_2^2, \quad \forall\boldsymbol{\alpha}\in\mathbb{R}^{pK} \text{ s.t. } \|\boldsymbol{\alpha}\|_\infty \leq 1,$$

and it is easy to verify that $\|\boldsymbol{\alpha}\|_2^2 \leq pK$ given that $\boldsymbol{\alpha}\in\mathbb{R}^{pK}$ and $\|\boldsymbol{\alpha}\|_\infty \leq 1$, which completes the proof.

## S.3 Proof of Proposition 3.2

The smoothness of $f_\mu(\boldsymbol{\beta};\lambda_{\mathrm{F}},\lambda_{\mathrm{Sim}})$ can be proved by applying the following Theorem 26.3 in Rockafellar [1970]. We start by the conjugate $\phi^*$ of $\phi(\boldsymbol{\alpha}) = \frac{1}{2}\|\boldsymbol{\alpha}\|_2^2$ defined on $\{\boldsymbol{\alpha} : \|\boldsymbol{\alpha}\|_\infty \leq 1\}$, which is given by

$$\phi^*(\boldsymbol{\beta}) = \sup_{\{\boldsymbol{\alpha}:\|\boldsymbol{\alpha}\|_\infty\leq 1\}} \left(\boldsymbol{\alpha}^T\boldsymbol{\beta} - \phi(\boldsymbol{\alpha})\right).$$

By plugging $\frac{\mathbf{D}_{\lambda_{\mathrm{F}},\lambda_{\mathrm{Sim}}}\boldsymbol{\beta}}{\mu}$ into the conjugate function, we have

$$\mu\phi^*\left(\frac{\mathbf{D}_{\lambda_{\mathrm{F}},\lambda_{\mathrm{Sim}}}\boldsymbol{\beta}}{\mu}\right) = \sup_{\{\boldsymbol{\alpha}:\|\boldsymbol{\alpha}\|_\infty\leq 1\}}\left(\boldsymbol{\alpha}^T\mathbf{D}_{\lambda_{\mathrm{F}},\lambda_{\mathrm{Sim}}} - \frac{\mu}{2}\|\boldsymbol{\alpha}\|_2^2\right) = f_\mu(\boldsymbol{\beta};\lambda_{\mathrm{F}},\lambda_{\mathrm{Sim}}).$$

Therefore, $f_\mu$ has the essentially smooth conjugate function (we can easily verify that $\phi(\boldsymbol{\alpha}) = \frac{1}{2}\|\boldsymbol{\alpha}\|_2^2$ defined on $\{\boldsymbol{\alpha} : \|\boldsymbol{\alpha}\|_\infty \leq 1\}$ is essentially convex) and thus it is a smooth function.

To obtain the gradient $\nabla f_\mu$, we apply Danskin's theorem. Let

$$\psi(\boldsymbol{\alpha},\boldsymbol{\beta}) = \boldsymbol{\alpha}^T\mathbf{D}_{\lambda_{\mathrm{F}},\lambda_{\mathrm{Sim}}}\boldsymbol{\beta} - \frac{\mu}{2}\|\boldsymbol{\alpha}\|_2^2.$$

Then,

$$f_\mu(\boldsymbol{\beta};\lambda_{\mathrm{F}},\lambda_{\mathrm{Sim}}) = \max_{\{\boldsymbol{\alpha}:\|\boldsymbol{\alpha}\|_\infty\leq 1\}}\psi(\boldsymbol{\alpha},\boldsymbol{\beta}).$$

Since $\{\boldsymbol{\alpha} : \|\boldsymbol{\alpha}\|_\infty \leq 1\}$ is a compact set, $f_\mu$ is continuous in both $\boldsymbol{\alpha}$ and $\boldsymbol{\beta}$, and it is convex in $\boldsymbol{\beta}$ for every $\boldsymbol{\alpha}$ such that $\|\boldsymbol{\alpha}\|_\infty \leq 1$. Under these three conditions, Danskin's theorem grants that $f_\mu$ is convex in $\boldsymbol{\beta}$. Moreover,

$$\nabla f_\mu(\boldsymbol{\beta};\lambda_{\mathrm{F}},\lambda_{\mathrm{Sim}}) = \frac{\partial}{\partial\boldsymbol{\beta}}\psi(\boldsymbol{\alpha}^*,\boldsymbol{\beta}) = \mathbf{D}^T_{\lambda_{\mathrm{F}},\lambda_{\mathrm{Sim}}}\boldsymbol{\alpha}^*,$$

since the set

$$\left\{\boldsymbol{\alpha}^* : \psi(\boldsymbol{\alpha}^*,\boldsymbol{\beta}) = \max_{\{\boldsymbol{\alpha}:\|\boldsymbol{\alpha}\|_\infty\leq 1\}}\psi(\boldsymbol{\alpha},\boldsymbol{\beta})\right\}$$

has a single element because $\psi$ is strongly convex in $\boldsymbol{\alpha}$.

## S.4 Proof of Proposition 3.3

$\boldsymbol{\alpha}^*$ can be attained by solving the following optimization problem

$$\max_{\boldsymbol{\alpha}}\ \boldsymbol{\alpha}^T\mathbf{D}_{\lambda_{\mathrm{F}}},\lambda_{\mathrm{Sim}}\boldsymbol{\beta} - \frac{\mu}{2}\|\boldsymbol{\alpha}\|_2^2 \ \text{ s.t. } \|\boldsymbol{\alpha}\|_\infty \leq 1,$$

which can be rewritten as the following minimization problem

$$\min_{\boldsymbol{\alpha}} \frac{\mu}{2}\|\boldsymbol{\alpha}\|_2^2 - \boldsymbol{\alpha}^T \mathbf{D}_{\lambda_{\mathrm{F}},\lambda_{\mathrm{Sim}}}\boldsymbol{\beta} \ \text{ s.t. } \|\boldsymbol{\alpha}\|_\infty \le 1.$$

It is equivalent to

$$\min_{\boldsymbol{\alpha}} \left\| \boldsymbol{\alpha} - \frac{\mathbf{D}_{\lambda_{\mathrm{F}},\lambda_{\mathrm{Sim}}}\boldsymbol{\beta}}{\mu} \right\|_2^2 \ \text{ s.t. } \|\boldsymbol{\alpha}\|_\infty \le 1,$$

whose optimal solution satisfies

$$\alpha_i^* = \begin{cases} d_i & \text{if } d_i \in [-1,1] \\ 1 & \text{if } d_i \in (1,\infty) \\ -1 & \text{if } d_i \in (-\infty,-1) \end{cases},$$

where $d_i = \left[\frac{\mathbf{D}_{\lambda_{\mathrm{F}},\lambda_{\mathrm{Sim}}}\boldsymbol{\beta}}{\mu}\right]_i$ is the $i$-th element of $\frac{\mathbf{D}_{\lambda_{\mathrm{F}},\lambda_{\mathrm{Sim}}}\boldsymbol{\beta}}{\mu}$. Note that solving the minimization problem is equivalent to finding a Euclidean projection of $\frac{\mathbf{D}_{\lambda_{\mathrm{F}},\lambda_{\mathrm{Sim}}}\boldsymbol{\beta}}{\mu}$ onto the unit $L_\infty$ ball.

## S.5 Proof of Proposition 3.4

From Theorem 3.1, with $\mu = \frac{2\delta}{pK}$ for the approximation accuracy $0 < \delta < \varepsilon$, we have

$$F(\boldsymbol{\beta}^{(t)}) - F(\boldsymbol{\beta}^{**}) \le \delta + \frac{2\|\boldsymbol{\beta}^{(0)} - \boldsymbol{\beta}^*\|_2^2}{t^2}\left(\frac{1}{4}\max\left\{\lambda_{\max}(\mathbf{X}^{kT}\mathbf{X}^k) : k = 1,\cdots,K\right\} + \frac{pK}{2\delta}\|\mathbf{D}_{\lambda_{\mathrm{F}},\lambda_{\mathrm{Sim}}}\|_2^2\right).$$

Thus, the number of iterations $t$ to achieve $F(\boldsymbol{\beta}^{(t)}) - F(\boldsymbol{\beta}^{**}) \le \varepsilon$, is bounded by

$$\sqrt{\frac{2\|\boldsymbol{\beta}^{(0)} - \boldsymbol{\beta}^*\|_2^2}{\varepsilon - \delta}\left(\frac{1}{4}\max\left\{\lambda_{\max}(\mathbf{X}^{kT}\mathbf{X}^k) : k = 1,\cdots,K\right\} + \frac{pK}{2\delta}\|\mathbf{D}_{\lambda_{\mathrm{F}},\lambda_{\mathrm{Sim}}}\|_2^2\right)},$$

which can be simplified to $\mathcal{O}\left(\sqrt{\frac{pK}{\delta(\varepsilon-\delta)}}\right)$.

## S.6 Proof of Theorem 4.1

To prove the theorem, it is sufficient to show that $\mathcal{V}_n(\mathbf{u}^1, \mathbf{u}^2) \to \mathcal{V}(\mathbf{u}^1, \mathbf{u}^2)$ as $n \to \infty$, where $\mathcal{V}_n(\mathbf{u}^1, \mathbf{u}^2)$ is defined in (S.4).

From Theorem 4.1, we can re-write $\mathcal{V}(\mathbf{u}^1, \mathbf{u}^2)$ as

$$\mathcal{V}(\mathbf{u}^1, \mathbf{u}^2) = g(\mathbf{u}^1, \mathbf{u}^2) + h(\mathbf{u}^1, \mathbf{u}^2)$$

where

$$g(\mathbf{u}^1, \mathbf{u}^2) = \mathbf{u}^{1T}\mathbf{W}^1 + \mathbf{u}^{2T}\mathbf{W}^2 + \frac{1}{2}\mathbf{u}^{1T}\mathbf{C}^1\mathbf{u}^1 + \frac{1}{2}\mathbf{u}^{2T}\mathbf{C}^2\mathbf{u}^2$$

and

$$h(\mathbf{u}^1, \mathbf{u}^2) =$$

$$\lambda_{\mathrm{F}}^{(0)}\Bigg[(\bar{\mathbf{X}}_0^1\mathbf{u}^1 - \bar{\mathbf{X}}_0^2\mathbf{u}^2)\mathrm{sign}(\bar{\mathbf{X}}_0^1\boldsymbol{\beta}^1 - \bar{\mathbf{X}}_0^2\boldsymbol{\beta}^2)\mathbb{I}(\bar{\mathbf{X}}_0^1\boldsymbol{\beta}^1 \neq \bar{\mathbf{X}}_0^2\boldsymbol{\beta}^2) + |\bar{\mathbf{X}}_0^1\mathbf{u}^1 - \bar{\mathbf{X}}_0^2\mathbf{u}^2|\mathbb{I}(\bar{\mathbf{X}}_0^1\boldsymbol{\beta}^1 = \bar{\mathbf{X}}_0^2\boldsymbol{\beta}^2) +$$

$$(\bar{\mathbf{X}}_1^1\mathbf{u}^1 - \bar{\mathbf{X}}_1^2\mathbf{u}^2)\mathrm{sign}(\bar{\mathbf{X}}_1^1\boldsymbol{\beta}^1 - \bar{\mathbf{X}}_1^2\boldsymbol{\beta}^2)\mathbb{I}(\bar{\mathbf{X}}_1^1\boldsymbol{\beta}^1 \neq \bar{\mathbf{X}}_1^2\boldsymbol{\beta}^2) + |\bar{\mathbf{X}}_1^1\mathbf{u}^1 - \bar{\mathbf{X}}_y^2\mathbf{u}^2|\mathbb{I}(\bar{\mathbf{X}}_1^1\boldsymbol{\beta}^1 = \bar{\mathbf{X}}_1^2\boldsymbol{\beta}^2)\Bigg] +$$

$$\lambda_{\mathrm{Sp}}^{(0)}\sum_{k=1}^{2}\sum_{j=1}^{p}\{u_j^k\mathrm{sign}(\beta_j^k)\mathbb{I}(\beta_j^k \neq 0) + |u_j^k|\mathbb{I}(\beta_j^k = 0)\} +$$

$$\lambda_{\mathrm{Sim}}^{(0)}\sum_{j=1}^{p}\{(u_j^1 - u_j^2)\mathrm{sign}(\beta_j^1 - \beta_j^2)\mathbb{I}(\beta_j^1 \neq \beta_j^2) + |u_j^1 - u_j^2|\mathbb{I}(\beta_j^1 = \beta_j^2)\}.$$

Let

$$\mathcal{V}_n(\mathbf{u}^1, \mathbf{u}^2) = g_n(\mathbf{u}^1, \mathbf{u}^2) + h_n(\mathbf{u}^1, \mathbf{u}^2) \tag{S.4}$$

where

$$g_n(\mathbf{u}^1, \mathbf{u}^2) = -\left\{\ell\left(\boldsymbol{\beta}^1 + \frac{\mathbf{u}^1}{\sqrt{n}}\right) - \ell(\boldsymbol{\beta}^1)\right\} - \left\{\ell\left(\boldsymbol{\beta}^2 + \frac{\mathbf{u}^2}{\sqrt{n}}\right) - \ell(\boldsymbol{\beta}^2)\right\},$$

and

$$h_n(\mathbf{u}^1,\mathbf{u}^2) = \lambda_{\mathrm{F}}\Bigg\{ \left|\bar{\mathbf{X}}_0^1\left(\boldsymbol{\beta}^1+\frac{\mathbf{u}^1}{\sqrt{n}}\right)-\bar{\mathbf{X}}_0^2\left(\boldsymbol{\beta}^2+\frac{\mathbf{u}^2}{\sqrt{n}}\right)\right| - \left|\bar{\mathbf{X}}_0^1\boldsymbol{\beta}^1-\bar{\mathbf{X}}_0^2\boldsymbol{\beta}^2\right| \tag{S.5}$$

$$+\left|\bar{\mathbf{X}}_1^1\left(\boldsymbol{\beta}^1+\frac{\mathbf{u}^1}{\sqrt{n}}\right)-\bar{\mathbf{X}}_1^2\left(\boldsymbol{\beta}^2+\frac{\mathbf{u}^2}{\sqrt{n}}\right)\right| - \left|\bar{\mathbf{X}}_1^1\boldsymbol{\beta}^1-\bar{\mathbf{X}}_1^2\boldsymbol{\beta}^2\right| \Bigg\}$$

$$+\lambda_{\mathrm{Sp}}\sum_{k=1}^{2}\sum_{j=1}^{p}\left\{\left|\beta_j^k+\frac{u_j^k}{\sqrt{n}}\right|-|\beta_j^k|\right\}$$

$$+\lambda_{\mathrm{Sim}}\sum_{j=1}^{p}\left\{\left|\left(\beta_j^1+\frac{u_j^1}{\sqrt{n}}\right)-\left(\beta_j^2+\frac{u_j^2}{\sqrt{n}}\right)\right|-|\beta_j^1-\beta_j^2|\right\}.$$

We first show $g_n(\mathbf{u}^1,\mathbf{u}^2)\to g(\mathbf{u}^1,\mathbf{u}^2)$, that is,

$$\ell_k\left(\boldsymbol{\beta}^k+\frac{\mathbf{u}^k}{\sqrt{n}}\right)-\ell_k(\boldsymbol{\beta}^k)\to(\mathbf{u}^k)^T\mathbf{W}^k+\frac{1}{2}(\mathbf{u^k})^T\mathbf{C}^k\mathbf{u^k}. \tag{S.6}$$

Following the arguments of Viallon et al. [2013], we apply Taylor series expansion on the left side of (S.6) which yields

$$\ell_k\left(\boldsymbol{\beta}^k+\frac{\mathbf{u}^k}{\sqrt{n}}\right)-\ell_k(\boldsymbol{\beta}^k)=\frac{\nabla\ell_k(\boldsymbol{\beta}^k)^T\mathbf{u}^k}{\sqrt{n}}+\frac{1}{2}\mathbf{u^k}^T\frac{\mathcal{I}(\boldsymbol{\beta}^k)}{n}\mathbf{u}^k+o_{\mathrm{P}}\left(\frac{1}{n}\right).$$

Here, $o_{\mathrm{P}}$ is the small $o$ with respect to the probability measure P. Assumption 1 ensures $\frac{1}{2}\mathbf{u}^{kT}\frac{\mathcal{I}(\boldsymbol{\beta}^k)}{n}\mathbf{u}^k\to\frac{1}{2}\mathbf{u}^{kT}\mathbf{C}^k\mathbf{u}^k$ and assumption 2 ensures $\nabla\ell_k(\boldsymbol{\beta}^k)^T\mathbf{u}^k/\sqrt{n}\to\mathbf{W}^{kk}$.

On the other hand, to show $h_n(\mathbf{u}^1,\mathbf{u}^2)\to h(\mathbf{u}^1,\mathbf{u}^2)$, we follow the arguments in Theorem 2 of Knight and Fu [2000]. For the first term of (S.5), we have

$$\begin{aligned}
&\lambda_{\mathrm{F}}^{(n)}\left\{\left|\bar{\mathbf{X}}_y^1\left(\boldsymbol{\beta}^1+\frac{\mathbf{u}^1}{\sqrt{n}}\right)-\bar{\mathbf{X}}_y^2\left(\boldsymbol{\beta}^2+\frac{\mathbf{u}^2}{\sqrt{n}}\right)\right|-\left|\bar{\mathbf{X}}_y^1\boldsymbol{\beta}^1-\bar{\mathbf{X}}_y^2\boldsymbol{\beta}^2\right|\right\}\\
&\quad=\lambda_{\mathrm{F}}^{(n)}\left\{\left|\bar{\mathbf{X}}_y^1\boldsymbol{\beta}^1-\bar{\mathbf{X}}_y^2\boldsymbol{\beta}^2+\frac{\bar{\mathbf{X}}_y^1\mathbf{u}^1-\bar{\mathbf{X}}_y^2\mathbf{u}^2}{\sqrt{n}}\right|-\left|\bar{\mathbf{X}}_y^1\boldsymbol{\beta}^1-\bar{\mathbf{X}}_y^2\boldsymbol{\beta}^2\right|\right\}\\
&\quad\to\lambda_{\mathrm{F}}^{(0)}(\bar{\mathbf{X}}_y^1\mathbf{u}^1-\bar{\mathbf{X}}_y^2\mathbf{u}^2)\mathrm{sign}(\bar{\mathbf{X}}_y^1\boldsymbol{\beta}^1-\bar{\mathbf{X}}_y^2\boldsymbol{\beta}^2)\mathbb{I}(\bar{\mathbf{X}}_y^1\boldsymbol{\beta}^1\neq\bar{\mathbf{X}}_y^2\boldsymbol{\beta}^2)+|\bar{\mathbf{X}}_y^1\mathbf{u}^1-\bar{\mathbf{X}}_y^2\mathbf{u}^2|\mathbb{I}(\bar{\mathbf{X}}_y^1\boldsymbol{\beta}^1=\bar{\mathbf{X}}_y^2\boldsymbol{\beta}^2),
\end{aligned}$$

as $n \to \infty$, for $y = 0, 1$. Similarly,

$$\lambda_{\mathrm{Sim}}^{(n)} \sum_{j=1}^{p} \left\{ \left| \left( \beta_j^1 + \frac{u_j^1}{\sqrt{n}} \right) - \left( \beta_j^2 + \frac{u_j^2}{\sqrt{n}} \right) \right| - \left| \beta_j^1 - \beta_j^2 \right| \right\}$$

$$= \lambda_{\mathrm{Sim}}^{(n)} \sum_{j=1}^{p} \left\{ \left| \beta_j^1 - \beta_j^2 + \frac{u_j^1 - u_j^2}{\sqrt{n}} \right| - \left| \beta_j^1 - \beta_j^2 \right| \right\}$$

$$\to \lambda_{\mathrm{Sim}}^{(0)} \sum_{j=1}^{p} \left\{ (u_j^1 - u_j^2) \mathrm{sign}(\beta_j^1 - \beta_j^2) \mathbb{I}(\beta_j^1 \neq \beta_j^2) + |u_j^1 - u_j^2| \mathbb{I}(\beta_j^1 = \beta_j^2) \right\},$$

as $n \to \infty$, for $k = 1, 2$. We also have

$$\lambda_{\mathrm{Sp}}^{(n)} \sum_{j=1}^{p} \left\{ \left| \beta_j^k + \frac{u_j^k}{\sqrt{n}} \right| - \left| \beta_j^k \right| \right\} \to \lambda_{\mathrm{Sp}}^{(0)} \sum_{j=1}^{p} \{ u_j^k \mathrm{sign}(\beta_j^k) \mathbb{I}(\beta_j^k \neq 0) + |u_j^k| \mathbb{I}(\beta_j^k = 0) \},$$

as $n \to \infty$.

We showed that $g_n(\mathbf{u}^1, \mathbf{u}^2) \to g(\mathbf{u}^1, \mathbf{u}^2)$ and $h_n(\mathbf{u}^1, \mathbf{u}^2) \to h(\mathbf{u}^1, \mathbf{u}^2)$ as $n \to \infty$. Thus, $\mathcal{V}_n(\mathbf{u}^1, \mathbf{u}^2) \to \mathcal{V}(\mathbf{u}^1, \mathbf{u}^2)$ as $n \to \infty$ as desired.

**Note:** The Theorem 4.1 is proved for the JFM with *L1* penalization. For a model defined with *L2* penalization, we can simply modify $h(\mathbf{u}^1, \mathbf{u}^2)$ as below.

$$\begin{aligned} h(\mathbf{u}^1, \mathbf{u}^2) = & \lambda_{\mathrm{F}}^{(0)} \Big[ (\bar{\mathbf{X}}_0^1 \mathbf{u}^1 - \bar{\mathbf{X}}_0^2 \mathbf{u}^2) \mathrm{sign}(\bar{\mathbf{X}}_0^1 \boldsymbol{\beta}^1 - \bar{\mathbf{X}}_0^2 \boldsymbol{\beta}^2) |\bar{\mathbf{X}}_0^1 \mathbf{u}^1 - \bar{\mathbf{X}}_0^2 \mathbf{u}^2| + \\ & (\bar{\mathbf{X}}_1^1 \mathbf{u}^1 - \bar{\mathbf{X}}_1^2 \mathbf{u}^2) \mathrm{sign}(\bar{\mathbf{X}}_1^1 \boldsymbol{\beta}^1 - \bar{\mathbf{X}}_1^2 \boldsymbol{\beta}^2) |\bar{\mathbf{X}}_1^1 \mathbf{u}^1 - \bar{\mathbf{X}}_y^2 \mathbf{u}^2| \Big] + \\ & \lambda_{\mathrm{Sp}}^{(0)} \sum_{k=1}^{2} \sum_{j=1}^{p} \{ u_j^k \mathrm{sign}(\beta_j^k) |u_j^k| + \lambda_{\mathrm{Sim}}^{(0)} \sum_{j=1}^{p} \{ (u_j^1 - u_j^2) \mathrm{sign}(\beta_j^1 - \beta_j^2) |u_j^1 - u_j^2| \}. \end{aligned}$$

Following Knight and Fu [2000] and the arguments above, we can show $\sqrt{n}$-consistency of the estimates obtained from a model with *L2* penalization. The consistency of estimates obtained form a model utilizing mixture of *L1* and *L2* penalization can be proved similarly.

## S.7 Computational Analysis

Figure S.1 displays JFM's empirical computational complexity against the number of features and sample sizes.

For the first experiment, we increased the number of covariates from 100 to 5,000 while fixing the sample size at 200 and 500 respectively. Figure S.1(a) shows that the JFM computation time is approximately $\mathcal{O}(p^{1.5})$, which is because Algorithm 1's per iteration complexity is linear in $p$ and its rate of convergence is proportional to $\sqrt{p}$. With $5,000$ features, JFM finishes in 9 seconds on one Intel Xeon Platinum 8268 Processor (2.90 GHz, 24 cores) and 32GB RAM.

We then varied the sample size to 7,000 (5:2 ratio between groups) while the number of features was fixed at 1,000. In Figure S.1(b), the computation time is approximately $\mathcal{O}(n)$ for $n > 1,000$, as shown in Proposition 3.5. For $n < 1,000$, the computation time is inversely proportional to $n$ because Problem (3) is ill-posed ($p > n$) and requires more iterations for convergence.

## S.8 Accelerated Smoothing Proximal Gradient Algorithm for SFM

Bechavod and Ligett [2017] suggested to use CVXPY [Diamond and Boyd, 2016] to solve the SFM optimization problem. Since the problem is convex, CVXPY it can easily be handled. However, CVXPY is equipped with a general quadratic optimization solver and it is not efficient enough to be scalable for high-dimensional problems. Here, we introduce a variant of Algorithm 1 to solve the SFM more efficiently. Consider the following SFM optimization problem:

$$\underset{\boldsymbol{\beta}}{\text{minimize}} \; -\ell(\boldsymbol{\beta};\mathbf{X},\mathbf{y}) + \lambda_{\mathrm{F}_0}\sum_{j<k}|(\bar{\mathbf{X}}_0^j - \bar{\mathbf{X}}_0^k)\boldsymbol{\beta}| + \lambda_{\mathrm{F}_1}\sum_{j<k}|(\bar{\mathbf{X}}_1^j - \bar{\mathbf{X}}_1^k)\boldsymbol{\beta}| + \lambda_{\mathrm{Sp}}\|\boldsymbol{\beta}\|_1. \quad \text{(S.7)}$$

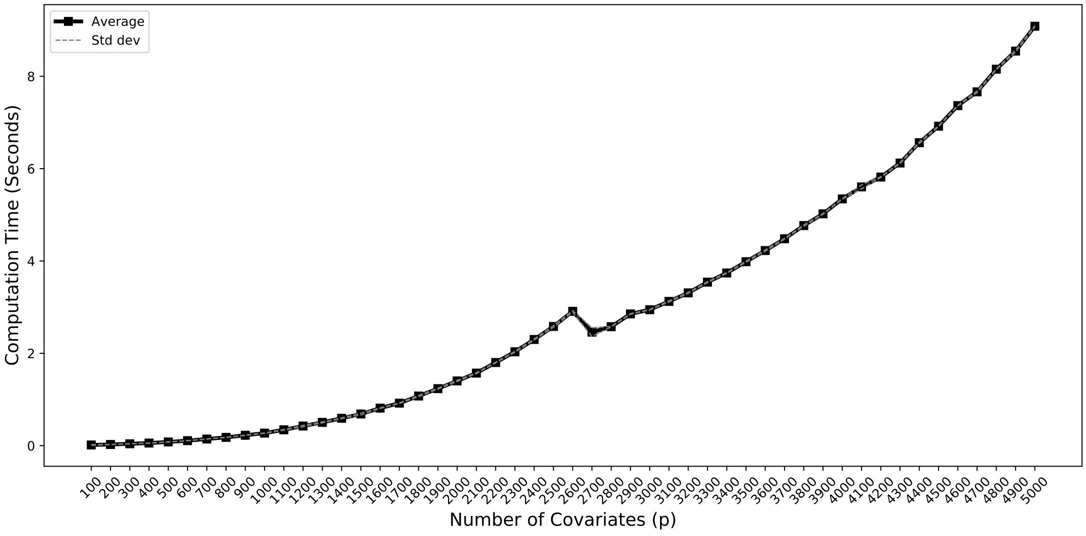


(a) Increasing Number of Covariates

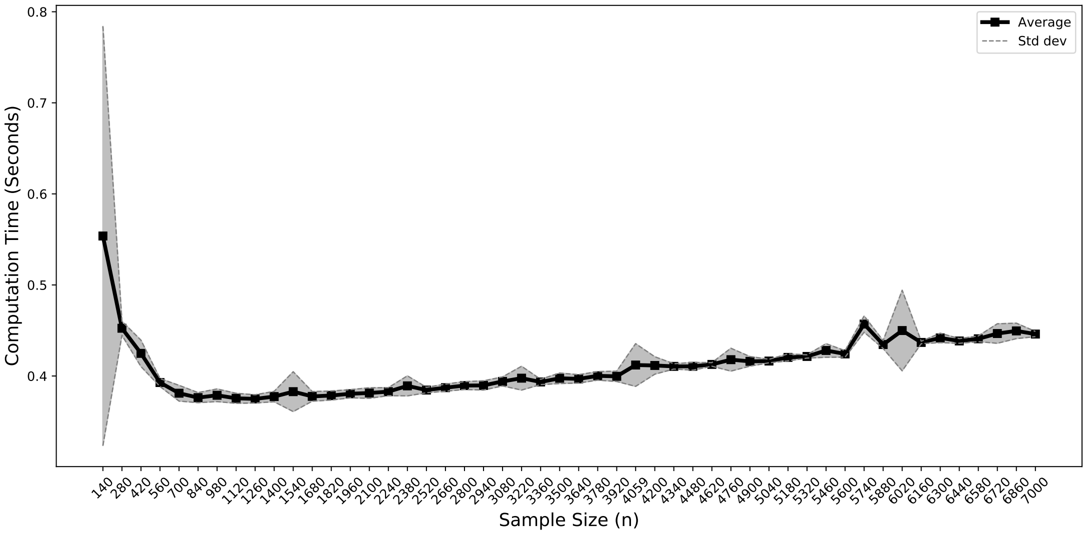


(b) Increasing Sample Size

Figure S.1: Experimental Results for Computational Analysis

Analogous to the matrix representation in Chapter 3, we can rewrite the objective function in matrix form:

$$\underset{\boldsymbol{\beta}}{\text{minimize}} - \ell(\boldsymbol{\beta}; \mathbf{X}, \mathbf{y}) + \|\mathbf{D}_{\lambda_\text{F}}\boldsymbol{\beta}\|_1 + \lambda_{\text{Sp}}\|\boldsymbol{\beta}\|_1,$$

where :

$$\mathbf{D}_{\lambda_\text{F}} = \begin{pmatrix} \lambda_{\text{F}_0}(\bar{\mathbf{X}}_0^1 - \bar{\mathbf{X}}_0^2) \\ \vdots \\ \lambda_{\text{F}_0}(\bar{\mathbf{X}}_0^{K-1} - \bar{\mathbf{X}}_0^K) \\ \lambda_{\text{F}_1}(\bar{\mathbf{X}}_1^1 - \bar{\mathbf{X}}_1^2) \\ \vdots \\ \lambda_{\text{F}_1}(\bar{\mathbf{X}}_1^{K-1} - \bar{\mathbf{X}}_1^K) \end{pmatrix}.$$

We can easily verify the Nesterov smooth approximation can be applied to approximate $\|\mathbf{D}_{\lambda_\text{F}}\boldsymbol{\beta}\|_1$ and Proposition 3.1, 3.2, and 3.3 hold. Therefore, Algorithm 2 solves the SFM optimization problem.

**Algorithm 2** Accelerated Smoothing Proximal Gradient Method for SFM

**Input:** Data $\mathbf{X}, \mathbf{y}$, hyperparameters $\lambda_{\mathrm{F}_0}, \lambda_{\mathrm{F}_1}, \lambda_{\mathrm{Sp}}$, $\epsilon$, $\mu$

**Output:** $\hat{\boldsymbol{\beta}}$ solving the Single Fairness optimization problem (S.7).

**Initialize:** $\boldsymbol{\beta}^{(0)} = \mathbf{0},\ \boldsymbol{\gamma}^{(0)} = \mathbf{0},\ s^{(0)} = 1$

Compute $L = \frac{1}{4}\lambda_{\max}(\mathbf{X}^T\mathbf{X}) + \mu^{-1}\|\mathbf{D}_{\lambda_{\mathrm{F}}}\|_2^2$

**for** $m \geq 1$ **do**

  $\boldsymbol{\alpha}^{(m)} = \boldsymbol{\gamma}^{(m-1)} - L^{-1}\left(-\nabla \ell\left(\boldsymbol{\gamma}^{(m-1)}\right) + \nabla f_\mu\left(\boldsymbol{\gamma}^{(m-1)}\right)\right)$

  $\boldsymbol{\beta}^{(m)} = \mathcal{S}\left(\boldsymbol{\alpha}^{(m)}; L^{-1}\lambda_{\mathrm{Sp}}\right)$

  **if** $\|\boldsymbol{\beta}^{(m)} - \boldsymbol{\beta}^{(m-1)}\|_2 \leq \epsilon$ **break**

  $s^{(m)} = \frac{1+\sqrt{1+4{s^{(m-1)}}^2}}{2}$

  $\boldsymbol{\gamma}^{(m)} = \boldsymbol{\beta}^{(m)} + \left(\frac{s^{(m-1)}-1}{s^{(m)}}\right)\left(\boldsymbol{\beta}^{(m)} - \boldsymbol{\beta}^{(m-1)}\right)$

  $m \leftarrow m + 1$

**end for**

$\hat{\boldsymbol{\beta}} \leftarrow \boldsymbol{\beta}^{(m)}$.

## S.9 Choice of the Evaluation Metrics in Selecting Hyperparameters in Cross-validations

The group-ignorant model, group-separate model, SFM, and JFM contain 1, $K$, 2, and $K + 2$ hyperparameters respectively. For every method, 5-fold cross-validation on the training dataset was used to determine the hyperparameters. For the vanilla models (group-separate and group-ignorant), the lasso penalty term was selected by optimizing cross-validation AUCs. For the fairness-aware models, we compared a series of evaluation metrics for selecting the hyperparameters in cross-validations, including group average of AUCs/accuracies (arithmetic mean, geometric mean, and harmonic mean), overall AUCs/accuracies on all samples ignoring group memberships, and the group average of

AUCs/accuracies subtracting the disparity of AUCs/accuracies (absolute differences and squared differences). Supplementary Figures S.2, S.3, and S.4 show the prediction performances in the test datasets with the optimal hyperparameters selected by various metrics. They demonstrate that the performances in the test datasets with the hyperparameters optimizing group-average AUCs in cross-validations were more optimal than those with the hyperparameters optimizing overall AUCs in cross-validations. Although the hyperparameters chosen to optimize group average of AUCs subtracting disparities provided better fairness performance in test datasets, it was often achieved by lowering the performance of the over-represented group. We also note that the hyperparameters optimizing AUC-based evaluation metrics generated more robust performances in test datasets than those optimizing threshold-based metrics such as accuracies and TPRs/TNRs. Therefore, the simulation results use the hyperparameters optimized by the harmonic mean of group-wise AUCs in cross-validations.

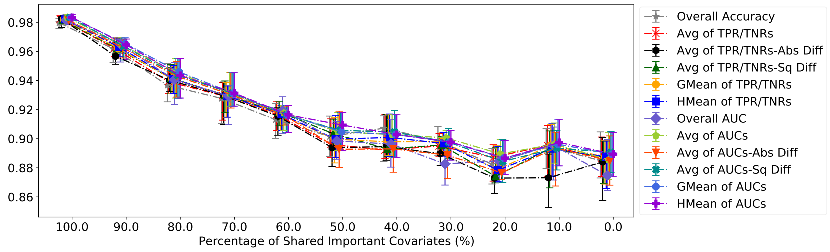


(a) AUC of the Under-represented Group

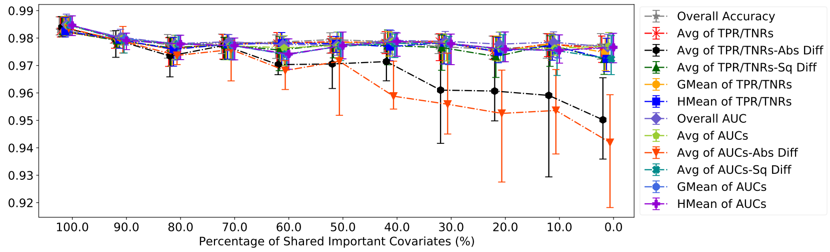


(b) AUC of the Over-represented Group

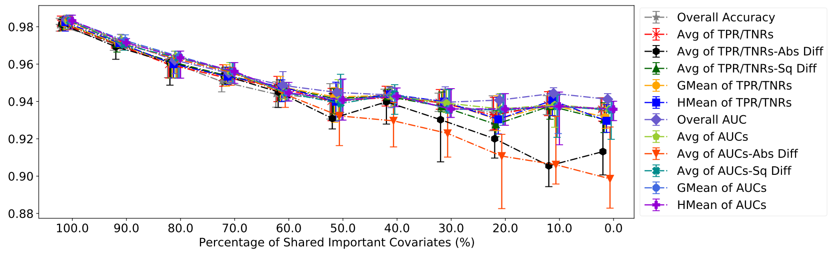


(c) Overall AUC

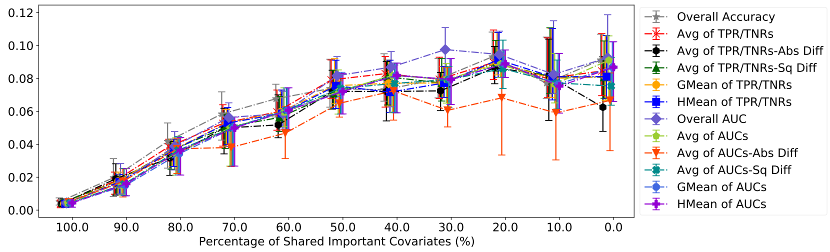


(d) Disparity of AUC

Figure S.2: Experimental Results for Evaluation Metrics on Scenario 1

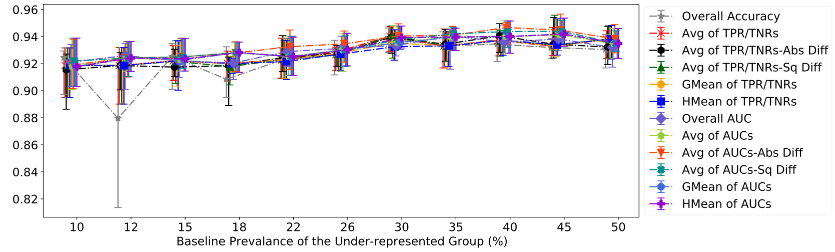


(a) AUC of the Under-represented Group

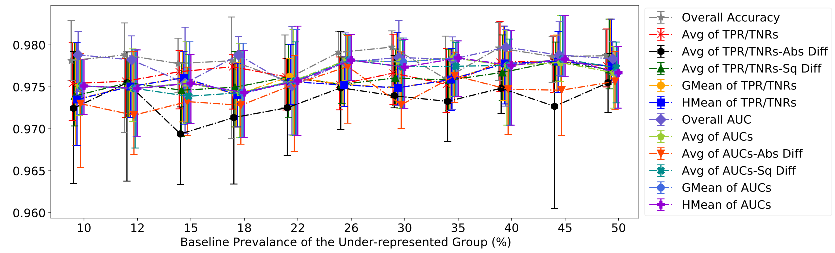


(b) AUC of the Over-represented Group

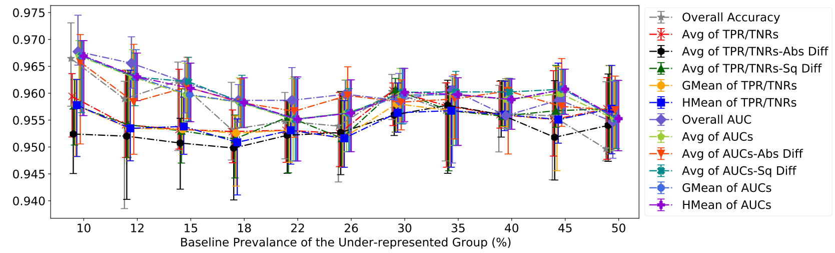


(c) Overall AUC

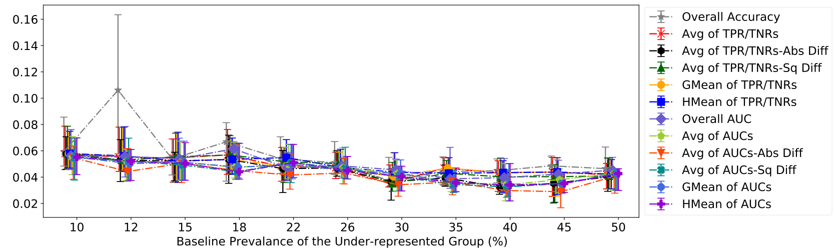


(d) Disparity of AUC

Figure S.3: Experimental Results for Evaluation Metrics on Scenario 2

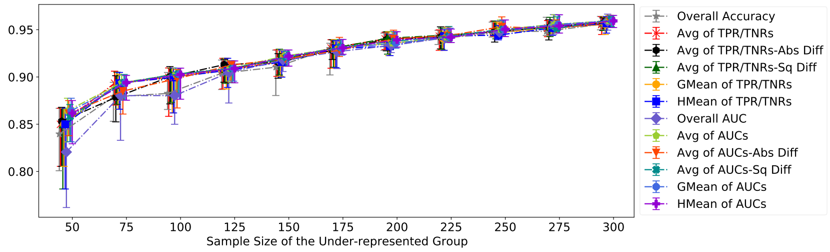


(a) AUC of the Under-represented Group

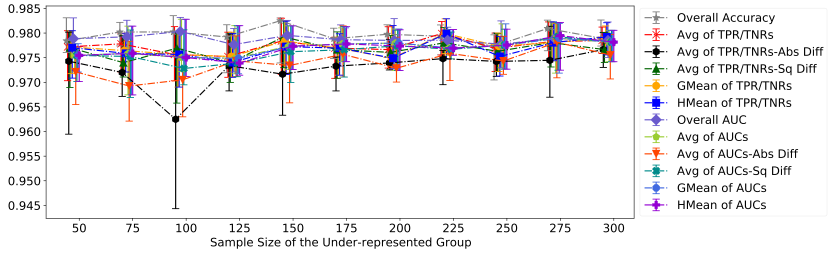


(b) AUC of the Over-represented Group

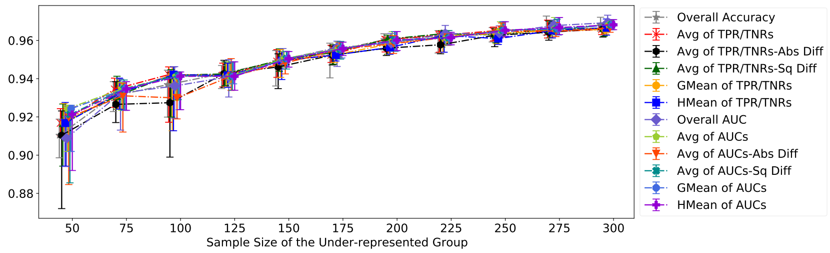


(c) Overall AUC

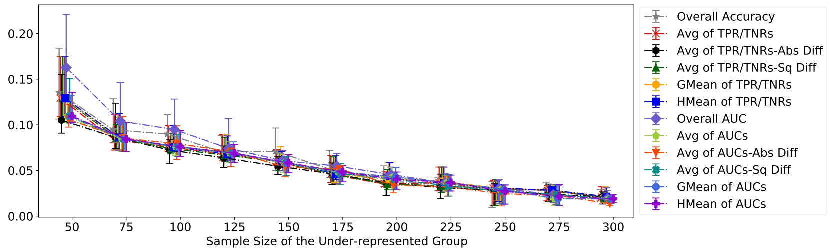


(d) Disparity of AUC

Figure S.4: Experimental Results for Evaluation Metrics on Scenario 3

## S.10 Average of TPR and TNR Plots for Simulation Study

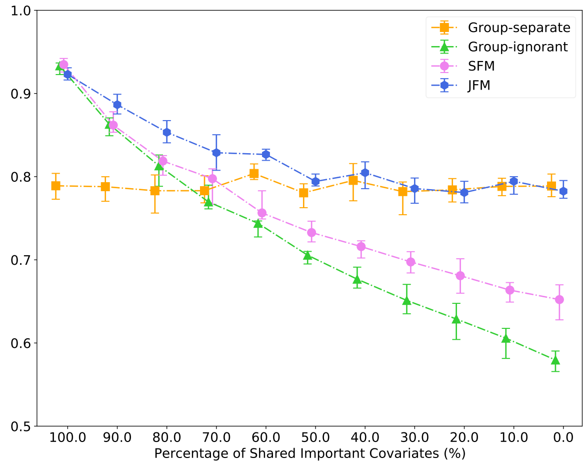


(a) Average of TPR and TNR of the Under-represented Group

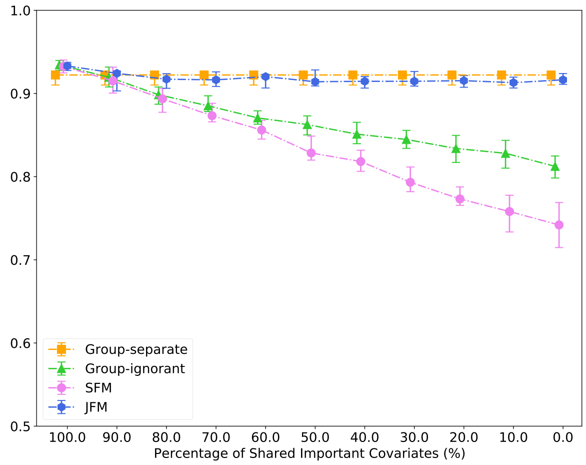


(b) Average of TPR and TNR of the Over-represented Group

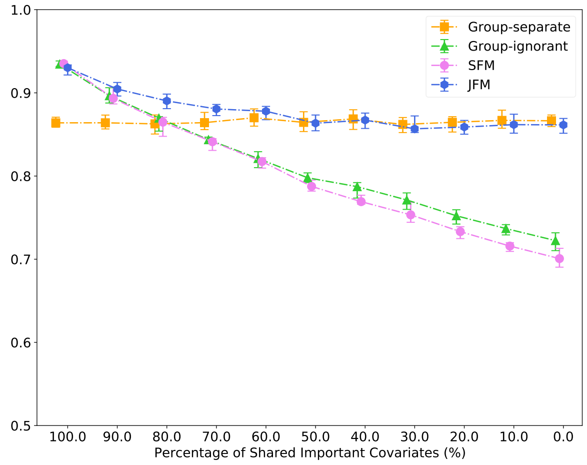


(c) Overall Average of TPR and TNR

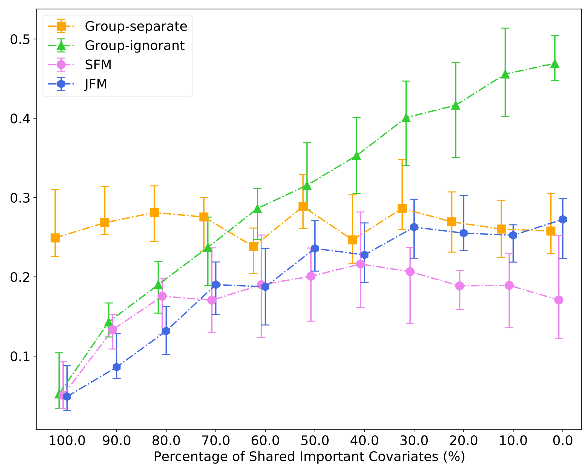


(d) Disparity of TPR and TNR

Figure S.5: Experimental Results for Scenario 1 (TPR + TNR)

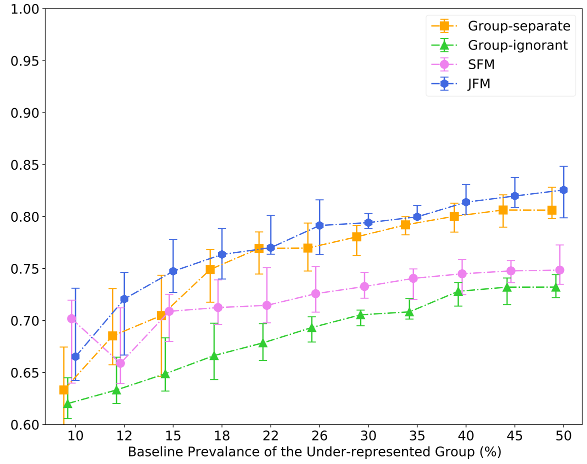


(a) Average of TPR and TNR of the Under-represented Group

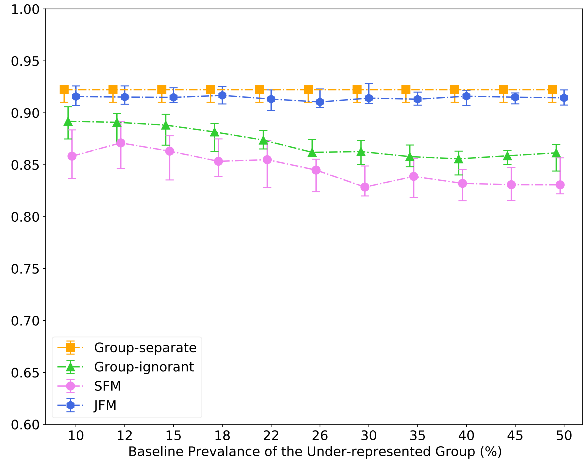


(b) Average of TPR and TNR of the Over-represented Group

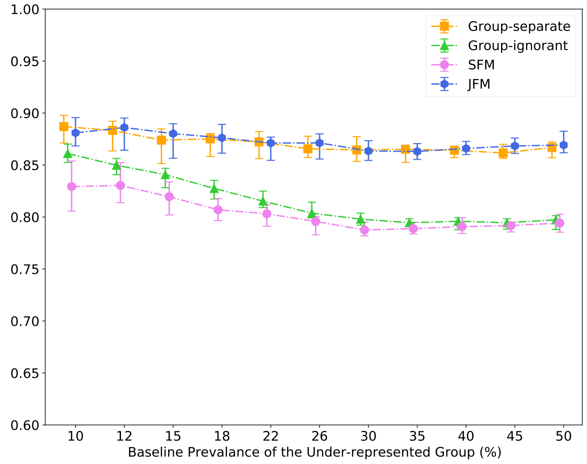


(c) Overall Average of TPR and TNR

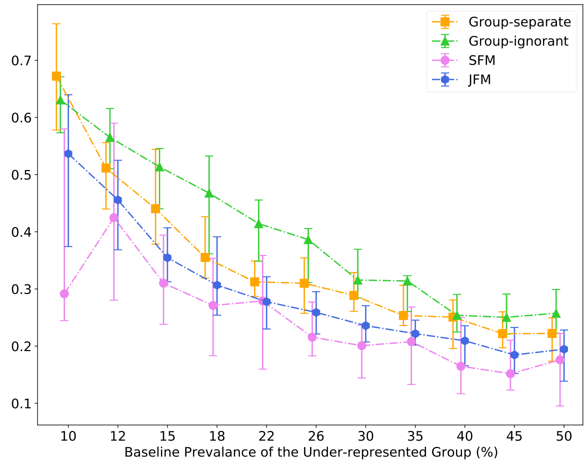


(d) Disparity of TPR and TNR

Figure S.6: Experimental Results for Scenario 2 (TPR + TNR)

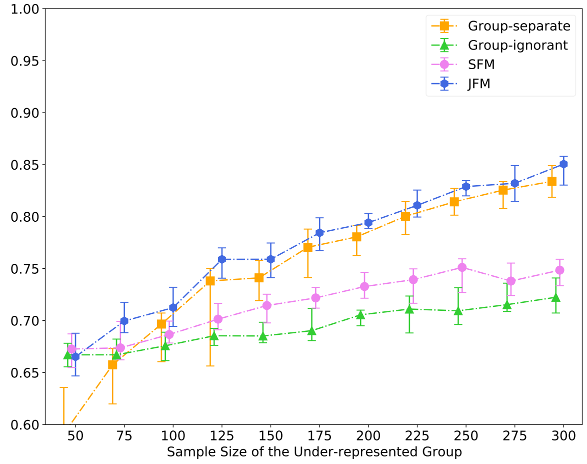


(a) Average of TPR and TNR of the Under-represented Group

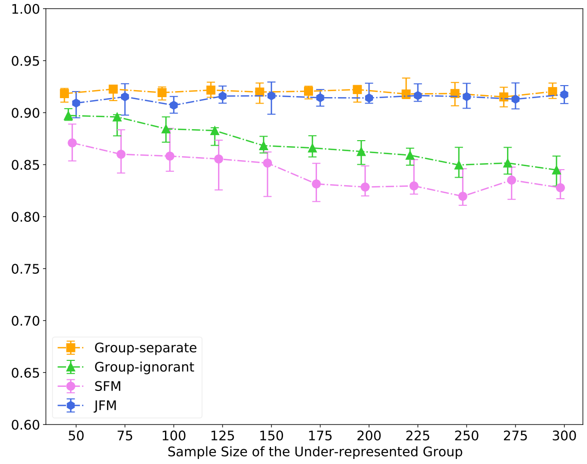


(b) Average of TPR and TNR of the Over-represented Group

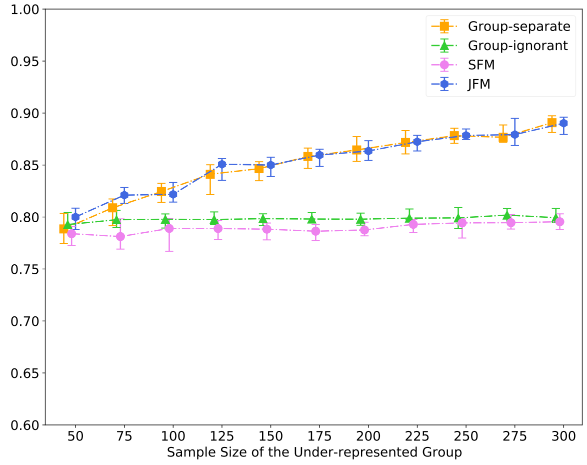


(c) Overall Average of TPR and TNR

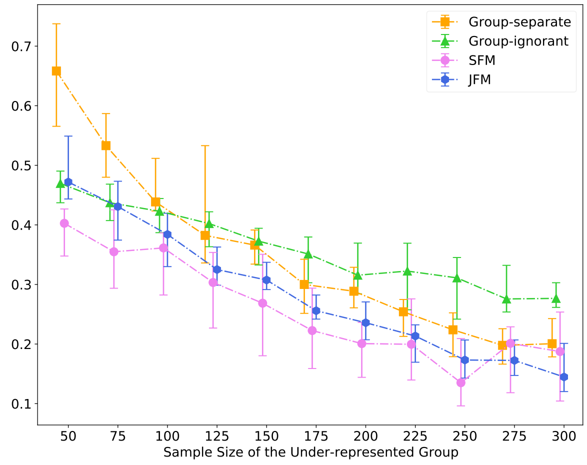


(d) Disparity of TPR and TNR

Figure S.7: Experimental Results for Scenario 3 (TPR + TNR)

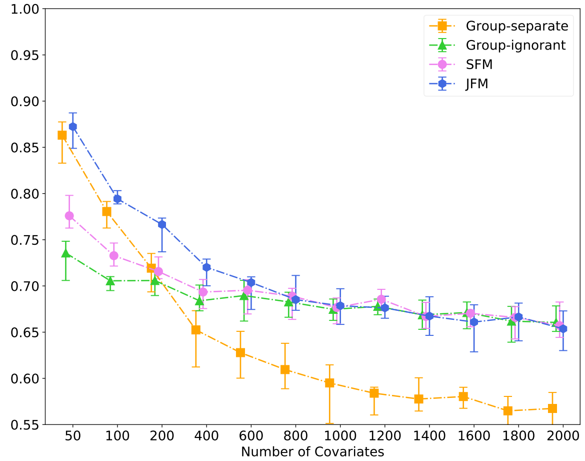


(a) Average of TPR and TNR of the Under-represented Group

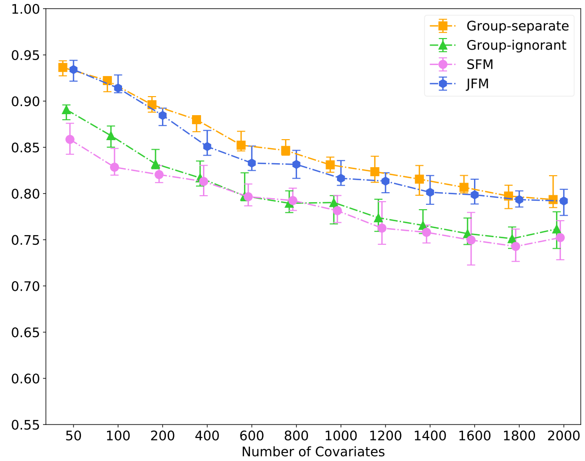


(b) Average of TPR and TNR of the Over-represented Group

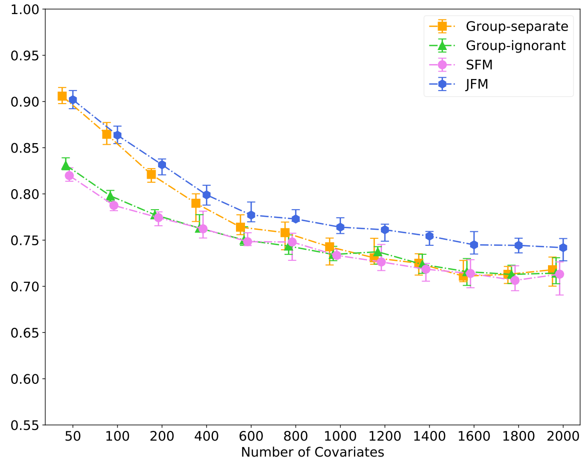


(c) Overall Average of TPR and TNR

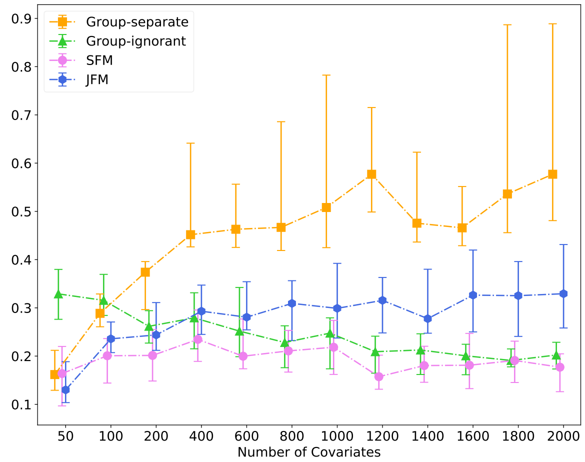


(d) Disparity of TPR and TNR

Figure S.8: Experimental Results for Scenario 4 (TPR + TNR)

## S.11 Additional Simulation Scenarios

Here, we present the results for additional simulation scenarios. The datasets are generated in the same way as in Section 5.

- In Scenario 1B, the number of non-zero coefficients of the under-represented group ranged from 20 to 40. The number of shared features fixed at 20, the baseline prevalence were 50% and 30% for the over and under-represented groups, respectively. The sample sizes were set at 500 and 200 for over and under-represented groups. The number of features were $p = 100$.
- In Scenario 2B, the baseline prevalence of the under-represented group ranged from 50% to 90% while the baseline event prevalence of the over-represented group was fixed at 50%.
- In Scenario 3B, samples size of the over-represented group ranged from 500 to 2500 with the sample size of the under-represented group fixed at 200.
- In Scenario 4B, the number of features $p$ ranged from 50 to 2,000. Everything is same with the Scenario 4, except that for each $p$, 30% of the features had non-zero coefficients.

As same as with the Section 5, we evaluated the methods on independent testing datasets under the same setups with large sample sizes (both 1000). AUC was used to evaluate the predictive performance of each model.

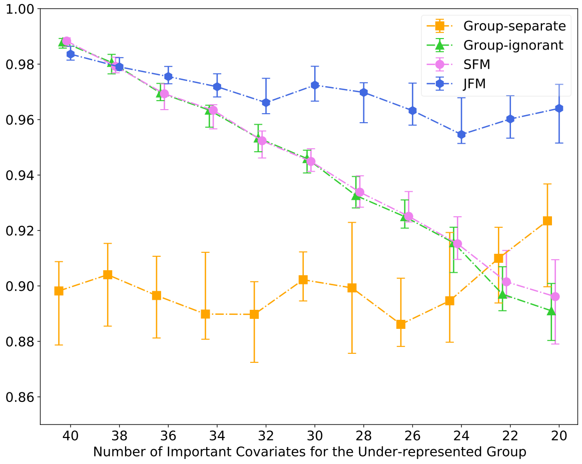


(a) AUC of the Under-represented Group

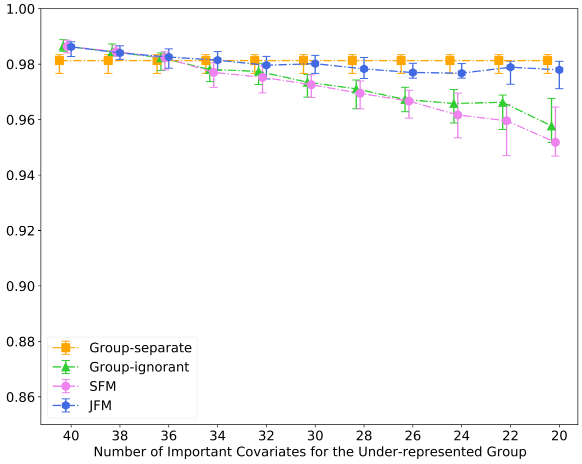


(b) AUC of the Over-represented Group

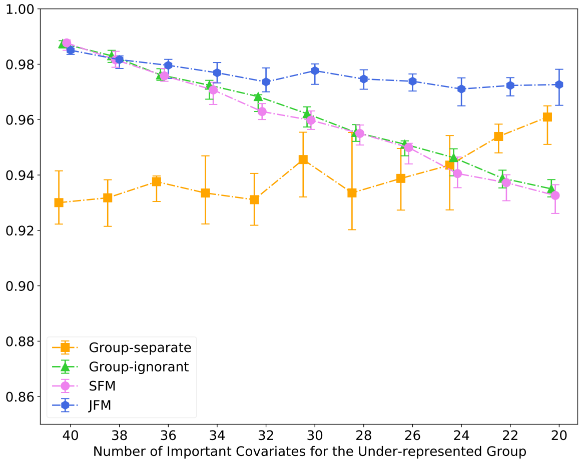


(c) Overall AUC

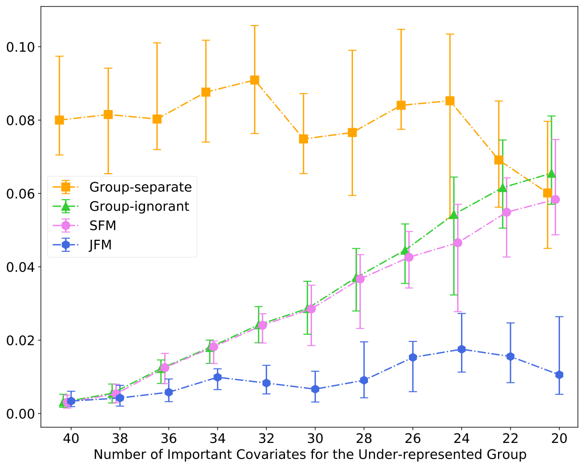


(d) Disparity of AUC

Figure S.9: Experimental Results for Scenario 1B

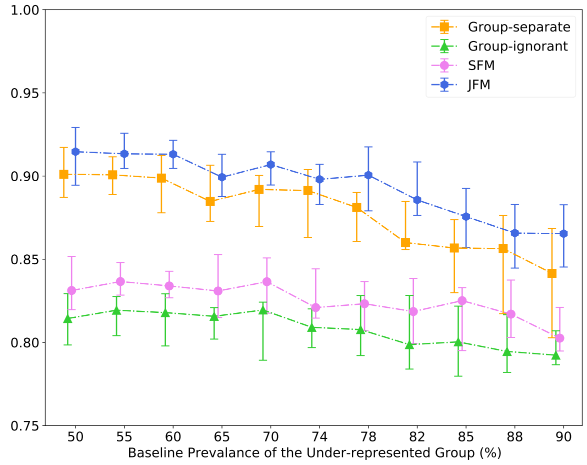


(a) AUC of the Under-represented Group

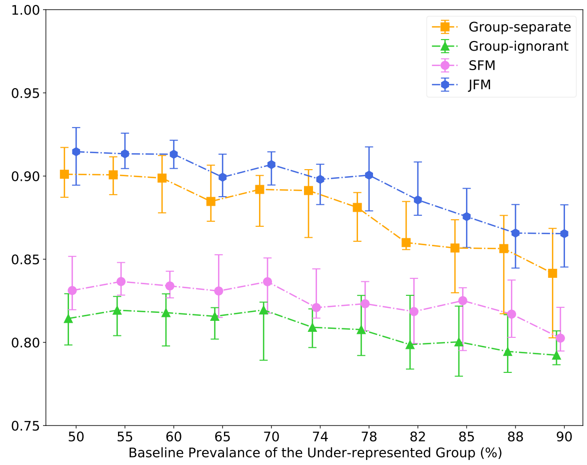


(b) AUC of the Over-represented Group

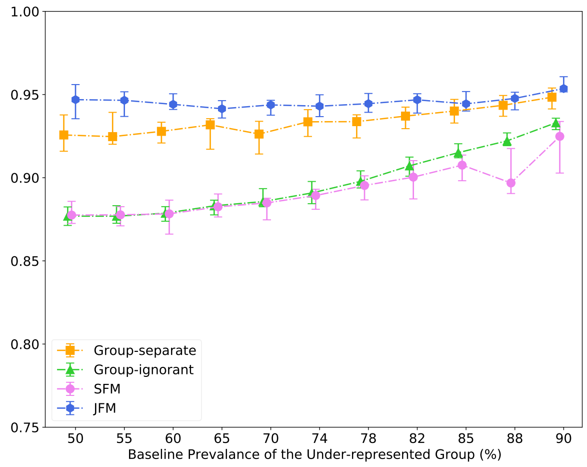


(c) Overall AUC

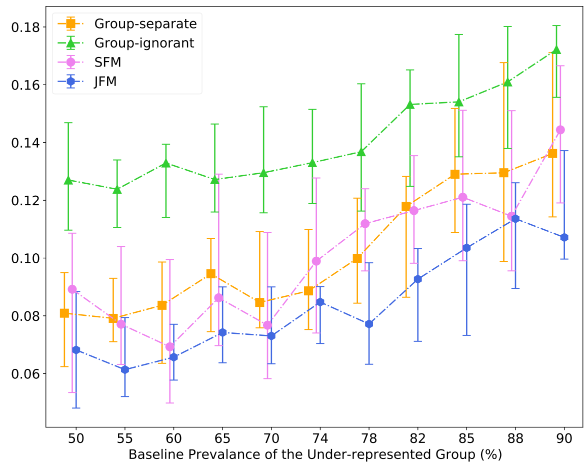


(d) Disparity of AUC

Figure S.10: Experimental Results for Scenario 2B

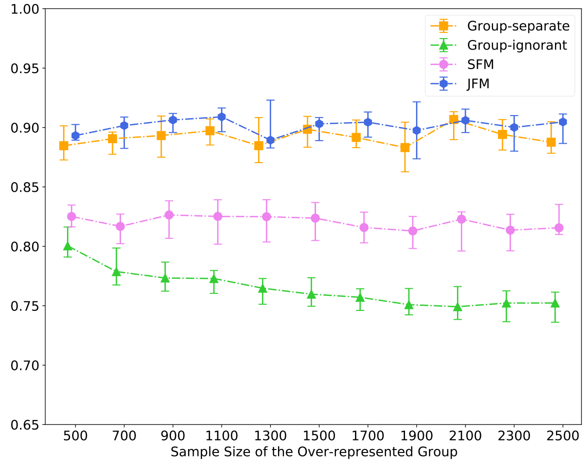


(a) AUC of the Under-represented Group

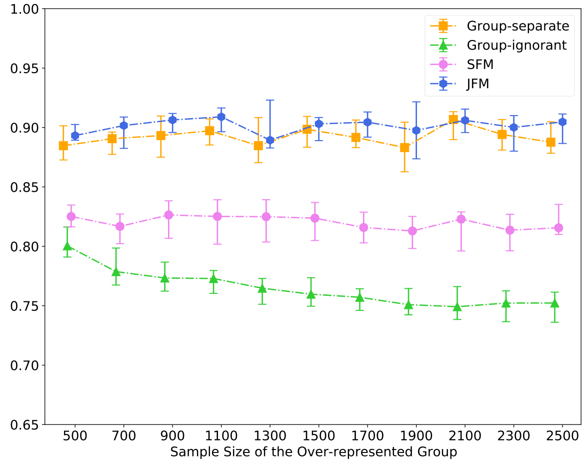


(b) AUC of the Over-represented Group

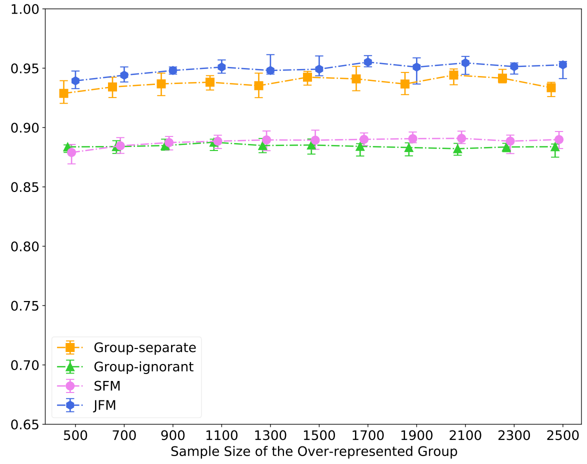


(c) Overall AUC

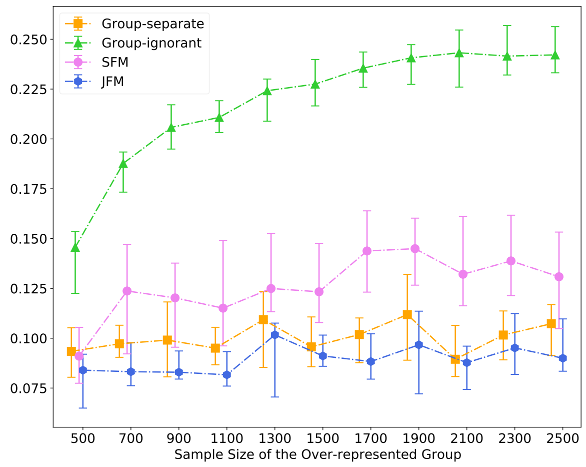


(d) Disparity of AUC

Figure S.11: Experimental Results for Scenario 3B

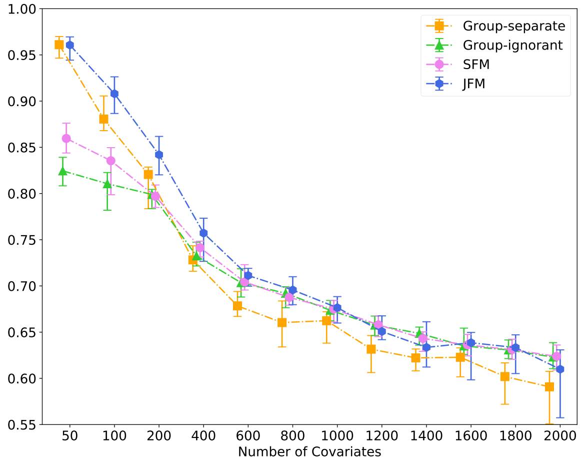


(a) AUC of the Under-represented Group

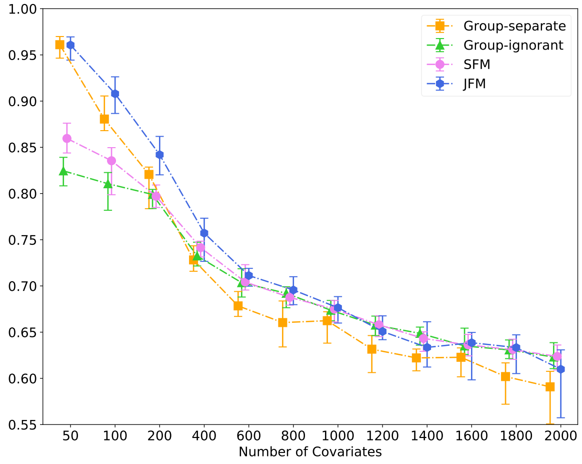


(b) AUC of the Over-represented Group

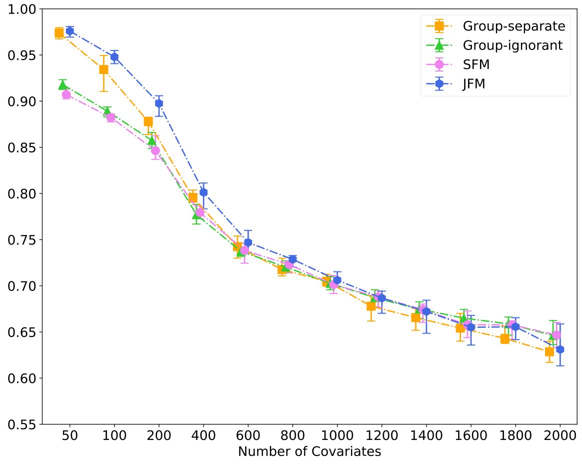


(c) Overall AUC

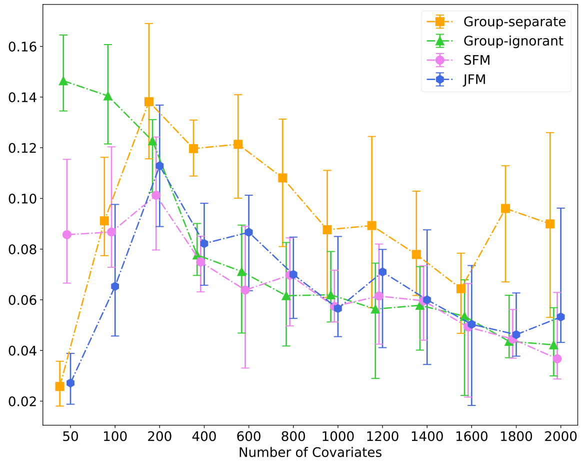


(d) Disparity of AUC

Figure S.12: Experimental Results for Scenario 4B

## S.12 Python Implementation

We provide a Python implementation to reproduce the simulation study results. The codes will be available at `https://github.com/hyungrok-do/joint-fairness-model`.

**Dependencies:**

- anaconda3 ($\geq$ 4.8.3)
- cython ($\geq$ 0.29.8)
- scipy ($\geq$ 1.6.2)
- numpy ($\geq$ 1.17.0)
- pandas ($\geq$ 1.2.4)
- matplotlib ($\geq$ 3.1.1)
- scikit-learn ($\geq$ 0.24.1)

**Install:** Users have to compile the enclosed cython source code (tested on Windows 10, macOS Catalina 10.15.7, and Red Hat Enterprise Linux 8.2.) After unzipping or cloning the git, type

```
python setup.py build_ext --inplace.
```

**Reproducing the results:** We provide shell/slurm scripts to run the repeated experiments to reproduce the results. For the results of scenarios 1 through 4 and the supplementary results scenarios 1B through 4B, use `run-simulation.sh` or `run-simulation.s`. To draw the plots, run `visualization-simulation-results.py`.

For the experiments for validation measures, execute `run-validation-measure.sh` or `run-validation-measure.s`. To draw the plots, run `visualization-validation-measures.py`.

Executing `experiment-computation-time-p.py` and `experiment-computation-time-n.py` will produce the Figure S.1 (a) and (b), respectively.

# References – Supplementary


Yahav Bechavod and Katrina Ligett. Penalizing unfairness in binary classification. *arXiv preprint arXiv:1707.00044*, 2017.

Xi Chen, Qihang Lin, Seyoung Kim, Jaime G Carbonell, Eric P Xing, et al. Smoothing proximal gradient method for general structured sparse regression. *The Annals of Applied Statistics*, 6(2):719–752, 2012.

Steven Diamond and Stephen Boyd. CVXPY: A Python-embedded modeling language for convex optimization. *Journal of Machine Learning Research*, 17(83):1–5, 2016.

Keith Knight and Wenjiang Fu. Asymptotics for lasso-type estimators. *Annals of statistics*, pages 1356–1378, 2000.

R Tyrrell Rockafellar. *Convex analysis*. Number 28. Princeton university press, 1970.

Vivian Viallon, Sophie Lambert-Lacroix, Holger Höfling, and Franck Picard. Adaptive generalized fused-lasso: Asymptotic properties and applications. 2013. doi: hal-00813281f.
